# Tailored Perception: Individuals’ Speech and Music Perception Strategies Fit Their Perceptual Abilities

**DOI:** 10.1037/xge0000688

**Published:** 2019-10-07

**Authors:** Kyle Jasmin, Fred Dick, Lori L. Holt, Adam Tierney

**Affiliations:** 1 Department of Psychological Sciences, Birkbeck, University of London, and Institute of Cognitive Neuroscience, University College London; 2 Department of Psychological Sciences, Birkbeck, University of London, and Department of Experimental Psychology, University College London; 3 Department of Psychology, Carnegie Mellon University; 4 Department of Psychological Sciences, Birkbeck, University of London

**Keywords:** amusia, duration, music, pitch, speech

## Abstract

Perception involves integration of multiple dimensions that often serve overlapping, redundant functions, for example, pitch, duration, and amplitude in speech. Individuals tend to prioritize these dimensions differently (stable, individualized perceptual strategies), but the reason for this has remained unclear. Here we show that perceptual strategies relate to perceptual abilities. In a speech cue weighting experiment (trial *N* = 990), we first demonstrate that individuals with a severe deficit for pitch perception (congenital amusics; *N* = 11) categorize linguistic stimuli similarly to controls (*N* = 11) when the main distinguishing cue is duration, which they perceive normally. In contrast, in a prosodic task where pitch cues are the main distinguishing factor, we show that amusics place less importance on pitch and instead rely more on duration cues—even when pitch differences in the stimuli are large enough for amusics to discern. In a second experiment testing musical and prosodic phrase interpretation (*N* = 16 amusics; 15 controls), we found that relying on duration allowed amusics to overcome their pitch deficits to perceive speech and music successfully. We conclude that auditory signals, because of their redundant nature, are robust to impairments for specific dimensions, and that optimal speech and music perception strategies depend not only on invariant *acoustic* dimensions (the physical signal), but on *perceptual* dimensions whose precision varies across individuals. Computational models of speech perception (indeed, all types of perception involving redundant cues e.g., vision and touch) should therefore aim to account for the precision of perceptual dimensions and characterize individuals as well as groups.

Perception often involves integrating cues from multiple sources and arriving at a categorical interpretation. For instance, in visual speech (seeing a face while hearing speech), cues from the face and voice are integrated to arrive at categorical percepts (McGurk & MacDonald, 1976). Cues are also integrated within a single sensory domain: when perceiving speech and music, individuals classify what they hear, mapping continuous spectral and temporal variation onto linguistic units, and tonal/rhythmic variation onto musical units. This process occurs at and among multiple putative levels simultaneously, as smaller units such as phonemes, syllables, and words are combined into larger structures such as phrases, clauses and sentences (Bentrovato, Devescovi, D’Amico, Wicha, & Bates, 2003; Elman, 2009; Lerdahl & Jackendoff, 1985; McMurray, Aslin, Tanenhaus, Spivey, & Subik, 2008; Utman, Blumstein, & Burton, 2000). Similarly for vision, visual features from (e.g.) the eyes and mouth are integrated to arrive at emotional categories (Du, Tao, & Martinez, 2014).

The cues that indicate a category can be subtle, and are often distorted or degraded by noise in the environment. In the case of speech perception, which requires precise and rapid detection of acoustic cues (Holt & Lotto, 2008), one might think that biologically based difficulties with auditory perception would also be a significant barrier. Individuals differ widely on many dissociable auditory skills, such as the degree of temporal and spectral resolution they can perceive (Kidd, Watson, & Gygi, 2007). However, it would seem that these difficulties have little effect on the overall success of speech perception: decades of research have consistently found that nonverbal auditory skills either do not correlate (or only weakly correlate) with speech perception ability in normal-hearing adults, and factor analyses consistently separate verbal and nonverbal sound perception into separate factors (Karlin, 1942; Kidd et al., 2007; Stankov & Horn, 1980; Surprenant & Watson, 2001; Watson & Kidd, 2002; Watson, Jensen, Foyle, Leek, & Goldgar, 1982).

Even an extreme deficit in a single aspect of perception can leave overall perception unimpaired. Individuals with congenital amusia (a disorder of pitch perception discussed in more detail below) do not report problems understanding spoken language (F. Liu, Patel, Fourcin, & Stewart, 2010). How can an individual both be severely impaired in one aspect of perception and yet still perceive successfully? The answer may lie in the nature of evolved communicative systems, which have specific properties that make them robust to perturbation. Two such properties are *redundancy*, the presence of multiple identical structures, such as repetitions of the same note sequence in birdsong, and *degeneracy* (Price & Friston, 2002; Hebets et al., 2016), which refers to the presence of multiple *different* features that perform the same overlapping function, such as a smile conveyed by cues from both the shape of the mouth and the eyes (Del Giudice & Colle, 2007).

In speech, multiple redundant cues within the acoustic signal often perform the same linguistic function (see next section). It has been theorized that this property of speech could make it robust to any isolated perceptual deficit (Patel, 2014), as well as environmental noise (Winter, 2014), but to our knowledge this has not been demonstrated empirically. Indeed, this principle may also apply to other forms of perception. In music, for example, individuals with weak perceptual ability for one dimension may rely on some other dimension (or combination of other dimensions) to understand musical structure. Integration of redundant cues from multiple senses, such as vision and touch (Helbig & Ernst, 2007) may also depend on the reliability of those particular senses. For the purposes of this article we will refer to the phenomenon by which multiple cues index the same category feature as *redundancy* (although biological systems theorists would call this *degeneracy*), and N.B. there are compensatory strategies besides redundancy/degeneracy that may assist with perceptual impairments as well, such as those involving top-down predictive processes (e.g., Aydelott, Baer-Henney, Trzaskowski, Leech, & Dick, 2012; Ganong, 1980; Wicha, Moreno, & Kutas, 2004).

## Structural Redundancy in Speech and Music

We have mentioned that speech is a highly redundant signal wherein a linguistic category may be conveyed by many acoustic cues. Indeed, the distinction between voiced and unvoiced consonants (e.g., *rapid* vs. *rabid*), for example, is conveyed by as many as 16 different cues (Lisker, 1986), the most prominent of these being voice onset time (VOT; i.e., the time lapsed prior to voicing onset) and fundamental frequency or pitch of the voice (abbreviated as F0; Haggard, Ambler, & Callow, 1970; Lisker, 1957; Massaro & Cohen, 1976). Redundancy is a widespread feature of speech that occurs across multiple time scales and applies to many different linguistic features. These include *syllable stress* (*PREsent* vs. *preSENT*), which is conveyed by increases in syllabic amplitude, duration, and F0 (Fear, Cutler, & Butterfield, 1995; Mattys, 2000), *linguistic focus* (*it was HER* vs. *it WAS her*) conveyed by increases in word duration and greater F0 variation (Breen, Fedorenko, Wagner, & Gibson, 2010; Chrabaszcz, Winn, Lin, & Idsardi, 2014), and *phrase structure* (*No dogs are here* vs. *No, dogs are here*) conveyed by F0 variation and increased duration for the syllable just before the phrase boundary (de Pijper & Sanderman, 1994; Streeter, 1978). Music shows similar redundancies. For instance, the boundaries of *musical phrases*—the smallest group of related adjacent units in music—are characterized by changes in pitch (a shift from low to high or high to low) and timing (a shift toward longer note durations; Palmer & Krumhansl, 1987).

How do individuals integrate redundant information across different dimensions? Prior research on speech perception focusing on the “average individual” or “average listener” (as aggregated over the behavior of groups of participants) has indicated that the multiple dimensions contributing to speech categorization are not perceptually equivalent (Breen et al., 2010; Haggard et al., 1970; Liu & Holt, 2015; Massaro & Cohen, 1976; McMurray & Jongman, 2011). That is to say, some acoustic dimensions more reliably and robustly signal speech category membership, and therefore carry relatively more perceptual weight “primary dimensions” whereas other dimensions are somewhat less diagnostic and carry relatively less perceptual weight “secondary dimensions”. For example, when making perceptual decisions about voiced and unvoiced consonant-vowels in clear speech (*rapid* vs. *rabid*), VOT of the initial consonant is primary, whereas F0 of the following vowel is secondary (Haggard et al., 1970; Massaro & Cohen, 1976, 1977). Conversely, for linguistic focus such as emphasis on a particular word in a sentence, F0 is primary whereas word duration is secondary (Breen et al., 2010).

However, a major issue with this approach is that seeking to characterize an average individual assumes that all listeners are able to use perceptual dimensions equally, which may not be the case. What if individuals use different strategies that depend on the constraints they are placed under? Indeed, perceptual strategies have been demonstrated to be malleable (for review see Holt, Tierney, Guerra, Laffere, & Dick, 2018). For example, when speech is presented in masking noise, VOT (a durational cue) is harder to detect, making F0 a more reliable cue to which consonant was spoken. This leads to a corresponding shift in cue weights: in noise, participants down-weight VOT cues and up-weight F0 cues (Holt et al., 2018; Winn, Chatterjee, & Idsardi, 2013). Furthermore, dimensional weights also change when the co-occurrence statistics of different dimensions are changed, as in a foreign accent. When VOT and F0 cues are presented in a manner contrary to their usual covariation in English (Idemaru & Holt, 2011; Liu & Holt, 2015), listeners down-weight reliance on the secondary (F0) dimension because it is no longer informative (Idemaru & Holt, 2011, 2014). In addition, experimentally increasing the variability of a given acoustic dimension (thereby making it less reliable) also leads to down-weighting of that dimension (Holt & Lotto, 2006) and categorization training with feedback also can lead to changes in dimensional weights (Francis, Baldwin, & Nusbaum, 2000; Francis, Kaganovich, & Driscoll-Huber, 2008; S.-J. Lim & Holt, 2011). Overall, these results suggest that listeners continuously monitor and assess the evolving relationships among acoustic dimensions and speech categories, and adjust perceptual weights of acoustic dimensions accordingly (Holt et al., 2018; Holt & Lotto, 2006; Toscano & McMurray, 2010; Massaro & Cohen, 1993; see Prince, 2014 for an example from the music domain). Given that systematically changing specific aspects of the physical signal (i.e., acoustics) to make them more or less reliable can affect cue weights, it follows that differences in reliability resulting from an individual’s own uniquely-developed perceptual system might result in different weighting strategies.

## Individual Differences in Dimensional Weighting in Perceptual Categorization

Large individual differences (Yu & Zellou, 2019) in perceptual weighting have been reported across a variety of phonetic contrasts, including lexical tone in Mandarin (Chandrasekaran, Sampath, & Wong, 2010), place of articulation (Hazan & Rosen, 1991), stop consonant length (Idemaru, Holt, & Seltman, 2012), and vowels (Kim, Clayards, & Goad, 2018). However, these differences tend to be stable rather than random, having been found to be similar across testing sessions (Idemaru et al., 2012; Kim et al., 2018; Kong & Edwards, 2016). These and many other studies have established that typically developed individuals use different (but consistent!) strategies when perceiving speech—some place much more weight upon certain sources of evidence than others—even when listeners share the same linguistic background.

What is poorly understood is the underlying cause of these differences. Considered in the context of the large individual differences in auditory perception reviewed above, an ideal strategy for one individual may be suboptimal for another due to differences in the reliability of perception for different dimensions. We hypothesize that is an important factor driving these individualized and stable perceptual strategies—variability in the relative precision of different perceptual dimensions across individuals. Applying this hypothesis to an extreme case, individuals with a severe perceptual deficit specific to a particular auditory dimension may learn, over time, that this dimension is not useful for them, and perceptually weight it less in categorization—*even in cases when changes in that dimension are easily perceptible*. They instead may strategically down-weight cues that are (for them) unreliable, an alternative route to effective perceptual categorization. Here we tested this hypothesis by investigating a population with a severe domain-general perceptual deficit for pitch: participants with congenital amusia.

## Speech Perception in Amusia

Congenital amusia provides an ideal test case for examining the consequences of deficits in the perception of a single dimension on perceptual weighting strategies. Amusia is a disorder affecting around 1.5% of the population (Peretz & Vuvan, 2017), which is characterized by problems detecting small changes in pitch (Hyde & Peretz, 2004; Vuvan, Nunes-Silva, & Peretz, 2015) with otherwise normal cognitive and perceptual skills. This impaired pitch perception (possibly caused by reduced conscious access to pitch information; i.e., *pitch agnosia*; Omigie & Stewart, 2011; Peretz, Brattico, Järvenpää, & Tervaniemi, 2009; Tillmann, Lalitte, Albouy, Caclin, & Bigand, 2016) leads to deficits in musical abilities such as singing and melody recognition (Ayotte, Peretz, & Hyde, 2002; Dalla Bella, Giguère, & Peretz, 2009) as well as linguistic abilities such as statement/question judgments and perception of tone-language speech in background noise, in controlled laboratory settings (Ayotte et al., 2002; Hutchins, Gosselin, & Peretz, 2010; Jiang, Hamm, Lim, Kirk, & Yang, 2010; Nan, Sun, & Peretz, 2010; Patel, Foxton, & Griffiths, 2005; Patel, Wong, Foxton, Lochy, & Peretz, 2008; Peretz et al., 2002; Vuvan et al., 2015). Asking amusics about their experiences with language, however, reveals a paradox: only 7% of amusics report problems with speech perception in everyday life (Liu et al., 2010). How is it that amusics show problems with speech perception in the laboratory but self-report being unaffected in ecologically valid settings? One possibility is that amusics compensate for their poor pitch fidelity by relying on other, redundant, acoustic cues in the speech signal. If so, this would mean that computational models of speech—and indeed vision, touch, and music perception, and so forth—should aim to predict the behavior of individuals as well as groups. This can be carried out by including terms that reflect the precision (or inversely, noise) relating to perceptual dimensions, in addition to the physical input.

## Experiment 1

In Experiment 1, we used amusia as a test case to examine whether impairment along a single perceptual dimension leads to a shift in how different sources of information are weighted in perceptual categorization. Two groups of participants (individuals with amusia and controls) categorized stimuli drawn from two-dimensional acoustic spaces that varied in the extent to which fundamental frequency of the voice (referred to as F0, a strong indicator of vocal pitch), as well as durational information, pointed toward one of two linguistic interpretations. In a *phonetic* paradigm, stimuli differed in the identity of a word-initial consonant (*beer* vs. *pier*) whereas in a *prosodic* paradigm, stimuli differed in the location of linguistic focus (*STUDY music* vs. *study MUSIC*). Crucially, the primary acoustic dimension for the phonetic experiment was durational in nature, but the primary dimension for the prosody paradigm was F0—the dimension that amusics hear with less fidelity. We hypothesized that if an individual’s perception of a primary dimension is unimpaired, they should have no need to shift reliance to other, redundant perceptual dimensions because the primary dimension provides a robust signal to category identity (Holt et al., 2018; Schertz, Cho, Lotto, & Warner, 2016; Wu & Holt, 2018). However, if perception of a primary dimension is indeed impaired, listeners may strategically down-weight it in favor of a secondary dimension for which perception is not impaired.

Experiment 1 tested the hypothesis that the extent to which individuals rely on particular acoustic dimensions in perceiving speech can be predicted by the relative informativeness of the dimensions (i.e., which one is primary) and the presence or absence of a perceptual deficit for that dimension. Participants assigned tokens of spoken language to linguistic categories based on a phonetic contrast for which the primary cue was related to duration (for which no participant had a perceptual deficit). We used two stimulus sets—a *phonetic* and a *prosodic* set—which fully crossed F0 and duration parameters. *Phonetic* perception was assessed using synthesized speech sounds differing in voicing (/b/ vs. /p/), which can be conveyed via differences in voice onset time (VOT; a duration-related cue) and fundamental frequency of the following vowel (F0; a pitch-related cue). As described above, for most listeners, VOT is the primary (dominant) dimension for /b/-/p/ categorization, whereas F0 is secondary (Haggard et al., 1970). We predict that performance on this task should therefore not differ between groups; none of the participants was impaired in perceiving the primary dimension, and could therefore rely upon it.

The same participants also completed a similar task that examined a *prosodic* contrast for which the primary dimension was, instead, F0 (related to pitch, for which amusics have a deficit) and duration was secondary. Here, categorization of prosodic focus was primarily conveyed via pitch accents (carried by F0) and, to a lesser extent, durational lengthening (Breen et al., 2010). That is, F0 (the dimension amusics are impaired at perceiving) was the primary dimension, and the duration dimension was secondary. Thus, we predict that they will perceptually weight F0 less than control participants *even across F0 differences that are easily perceptible for amusic participants,* based on their pitch discrimination thresholds. This would indicate that any group differences in perceptual cue weighting between amusics and controls reflect differences in categorization strategy rather than simply an inability to detect the cues.

Both stimulus sets fully sampled an acoustic space created by sampling the orthogonal duration and F0 dimensions with equal probability. Participants therefore heard some stimuli that clearly belonged to one category or the other, and other stimuli that were perceptually ambiguous. This allowed us to measure the perceptual weight of the F0 and duration dimensions separately for the phonetic and prosodic paradigms of the experiment (Holt & Lotto, 2006), for both the amusic and control groups. Critically, we confirmed with psychophysical testing that F0 differences among our stimuli were large enough to be perceptible *even to amusic participants*. This ensured amusics could potentially use F0 information in the task.

### Method

#### Participants

Sixteen amusics (10 F, age = 60.2 ± 9.4) and 15 controls (10 F, age = 61.3 ± 10.4) were recruited from the U.K. and were native British English speakers with the exception of one amusic whose native language was Finnish but acquired English at age 10. (This participant was excluded from the Linguistic Phrase and Focus Test analyses of Experiment 2.) Audiometric thresholds were measured at left and right ears at octaves between 500 and 8,000 Hz for all control participants and all but one amusic participant; the two groups did not differ on hearing thresholds, *t*(28) = 1.33, *p* = .2. Experiment 1’s sample consisted of 11 amusics (6 F, age = 59.3) and 11 controls (8 F, age = 60.4) because, as it was run online, fewer participants had the equipment necessary to take part. All participants gave informed consent and ethical approval was obtained from the ethics committee for the Department of Psychological Sciences, Birkbeck, University of London. Participants were compensated £10 per hour of participation. Amusia status was obtained using the Montreal Battery for the Evaluation of Amusia (MBEA), in which participants heard pairs of short melodic phrases and rhythms across five subtests—Contour, Scale, Interval, Rhythm, Meter, and Musical Memory—and judged whether each pair were identical or different. Participants with a composite score (summing the Scale, Contour and Interval tests scores) of 65 or less were classified as amusic (Peretz, Champod, & Hyde, 2003). Most of the amusic participants (15) were recruited and had their amusia status confirmed previously by the Music, Mind, and Brain group at Goldsmiths University of London, and one was recruited online through circulation of an MBEA scale test that was administered through a web browser. No participant in either group had more than 5 years of musical training (amusic mean years 0.75; control mean years 1.35).

#### Phonetic cue weighting

Each phonetic block consisted of repetitions of a single word, spoken by a female American English speaker, that varied from “beer” (IPA: /bɪɹ/) to “pier” (IPA: /pɪɹ/) along two orthogonal acoustic dimensions. Praat 5.0 (Boersma, 2002) was used to alter original recordings of the words *beer* and *pier* such that the voice onset time (VOT) ranged from −5 ms to 15 ms in 5-ms increments. The F0 onset frequency of the vowel was manipulated manually in Praat such that it varied from 200 Hz to 320 Hz in 30 Hz increments. The F0 remained at this frequency for 80 ms, after which it decreased linearly to 180 Hz over the following 150 ms. Consistent with the distributional regularities of English, shorter VOT and lower initial F0 sounded more like /b/, whereas longer VOT and higher initial F0 sounded like /p/ (Idemaru & Holt, 2011). A full description of the stimulus creation methods can be found in Idemaru and Holt (2011). Each of the five F0 levels was fully crossed with each of the five VOT (duration) levels to make 25 stimuli. The stimulus set can be found in the online supplemental materials.

To ensure that the intervals along the F0 dimension were large enough for amusics to detect, we measured the median F0 of the vowel following the initial stop consonant for each of the five levels of F0. The vowel F0 for the five levels was 200, 230, 260, 290, and 320 Hz, with a mean difference between adjacent F0 levels of two semitones. This exceeded the largest pitch discrimination threshold across all participants, which was less than 1.5 semitones (see Experiment 2).

#### Prosodic cue weighting

Linguistic focus materials were constructed from combinations of two phrases, “Dave likes to STUDY music” and “Dave likes to study MUSIC” (stress indicated in uppercase). These phrases were spoken by an actor who read two compound sentences with an intervening conjunction, for example, “Dave likes to STUDY music, but he doesn’t like to PLAY music” and “Dave likes to study MUSIC, but he doesn’t like to study HISTORY.” The actor was asked to place contrastive accents to emphasize the capitalized words. These recordings were then cropped, leaving only the initial phrases listed above. Using STRAIGHT software (Kawahara & Irino, 2005), the two versions were manually time aligned. This was done by marking corresponding anchor points in the two sound files. (N.B. time-alignment does not result in output morphs being of the same durational length).

We then produced a set of 49 different stimuli by varying the extent to which F0 and durational information supported one versus the other focus interpretation. We then adjusted the morphing level for the temporal and F0 dimensions to synthesize intermediate versions of this spoken sentence. The value of morphing level can be interpreted as the relative contribution of one recording relative to another for a given auditory dimension. For instance, if a recording was synthesized with an F0 morphing level of 0%, and a temporal morphing level of 100%, that would mean that the F0 contour for the synthesized speech came entirely from the “STUDY music” recording, whereas the temporal duration information came entirely from the “study MUSIC” recording. If both the F0 and temporal morphing levels were set to 50%, that would mean that the F0 and durational aspects of the synthesized recording would reflect the midpoint, with F0 and duration information being halfway between both of the endpoint recordings. For both phonetic and prosodic stimulus sets, loudness did not vary across stimuli as only F0 and duration were manipulated.

**Figure 1 fig1:**
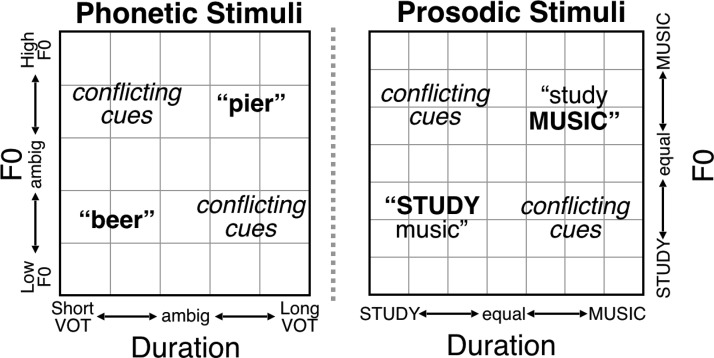
Schematic depiction of Phonetic and Prosodic stimulus spaces. In each stimulus set, a different linguistic interpretation was cued, to varying degrees, by the F0 contour and the duration of elements. Sometimes F0 and duration were indicative of the same interpretation, (upper right and bottom left corners) and other times the cues conflicted (upper left and bottom right). For the phonetic stimuli, five levels of an initial F0 excursion of the vowel (a pitch-related cue) and five levels of voice onset time (a duration cue) were crossed to create a phonetic stimulus space (Idemaru & Holt, 2011). For prosodic stimuli, seven morphing levels of F0 and seven of duration between *STUDY music* and *study MUSIC* were varied independently to create the stimulus space.

In the present experiment, the duration and F0 information disambiguating the focused word varied from 0% to 100% morphing levels for F0 and for duration, in 17% increments (0%, 17%, 33%, 50%, 67%, 83%, 100%). Because the dimensions were fully crossed, some combinations of F0 and duration cued an interpretation jointly, others conflicted, and tokens near the center of the space were more perceptually ambiguous (see Figure 1). Examples of the stimuli are provided in online supplemental materials.[Fig-anchor fig1]

To ensure that the intervals along the F0 dimension were large enough that amusics would be capable of detecting them, we measured the median fundamental frequency (F0) of the words *study* and *music* in Praat (Boersma, 2002; averaged across the entire word) for each of the seven levels of F0 morphing, then calculated the difference in F0 in semitones between the two words for each level. The differences in F0 between *study* and *music*, measured at the first vowel, at each of the seven F0 levels (negative values reflecting higher F0 on *music* than *study*) were −8.5, −5.0, −2.1, 0.6, 3.4 5.7, and 8.1 semitones. The difference in F0 between adjacent levels was always greater than two semitones. Because amusics are capable of detecting changes of two semitones and greater just as well as control participants (Hyde & Peretz, 2004; Mignault Goulet, Moreau, Robitaille, & Peretz, 2012; Moreau, Jolicœur, & Peretz, 2013; Peretz, Brattico, & Tervaniemi, 2005), and given that all of our participants had pitch discrimination thresholds of less than 1.5 semitones (see Experiment 2), we assume that the amusic participants in this study were capable of detecting the difference between stimuli varying along the F0 dimension. As a result, this paradigm is appropriate for investigating whether amusics perceptually weight potentially informative F0 information in phonetic and prosodic categorization.

#### Procedure

##### Pitch and duration thresholds

For all participants (Experiments 1 and 2, see below), pitch difference and duration difference thresholds were obtained in the laboratory with Maximum Likelihood Procedure (MLP; Grassi & Soranzo, 2009), an adaptive thresholding procedure based on the maximum likelihood method. Participants completed 3 blocks of 30 trials for both the pitch and duration threshold tests. On each trial participants heard three complex tones in a three-alternative forced-choice (3AFC) design. All complex tones comprised a fundamental frequency plus three harmonics, with 10-ms onset and offset cosine gates. For the pitch test, participants heard three complex tones that were 270 ms in duration. Two of these had an F0 of 330 Hz and one had a slightly higher F0 (with F0 difference adaptively determined via the MLP procedure). The order of presentation of the higher-F0 tone was randomized (first, second or third position). Participants indicated the temporal position of the higher tone by pressing keys 1, 2, or 3 on a keyboard.

For the duration test, participants heard three complex tones (F0 = 330 Hz). Two of these were 270 ms in duration and one was slightly longer, with the longer tone appearing randomly in any position and participants indicating its position by pressing, 1, 2, or 3 on the keyboard. The threshold was calculated as the pitch or duration value that led to correct responses on 80.9% of trials on a block-by-block basis. For both the duration and pitch threshold tests, the median threshold value across the three blocks was extracted for statistical analysis.

##### Speech-in-noise threshold

Participants’ ability to perceive speech in the presence of background noise was also assessed; this skill is known to be impaired in tone-language speaking amusics (F. Liu, Jiang, Wang, Xu, & Patel, 2015) but has not been examined in speakers of nontonal languages, who may rely more on duration cues when perceiving speech-in-noise (Lu & Cooke, 2009). Participants completed a speech-in-noise test, adapted from Boebinger et al. (2015). On each trial, a participant was presented with a short sentence from the Bamford-Kowal-Bench (BKB) corpus (Bench, Kowal, & Bamford, 1979) spoken by a female talker in the presence of competing background voices (multitalker babble). Maskers were presented at a constant level (0 dB) across trials while the loudness of the target voice was varied. Participants verbally repeated what they heard to the experimenter, so far as they were able. The experimenter had a predetermined list of three key words for each sentence and scored each trial by indicating how many of the three key words the participant had spoken on each trial (for a score of 0, 1, 2, or 3). The test adapted the signal-to-noise level using a one up, one down staircase procedure. The target level was varied first with an 8-dB step. The step reduced to 6-dB after the first reversal, 4-dB after the second reversal, and 2-dB after the third reversal. The procedure ended after six reversals at the smallest step size and the outcome measure was reported as the mean SNR level visited at the smallest step size.

##### Cue-weighting

The experiment began with instructions to listen to each item and classify whether the initial consonant was *B* or *P* (for the phonetic task) or whether the emphasis resembled *STUDY music* or *study MUSIC* (prosodic task). Participants heard one example of *beer*, *pier*, *study MUSIC*, and *STUDY music* during the instruction portion of the experiment. Responses were made by clicking a mouse to indicate one of two buttons positioned near the center of the screen (*B*/*STUDY music* on left; *P*/*study MUSIC* on right). Each phonetic block contained 50 trials (2 measurements at each of the 25 combinations of F0 and duration). Each focus block contained 49 trials (1 measurement at each of the 49 F0 and duration combinations). Each participant completed 20 blocks in total: 10 phonetic blocks (500 trials total, 20 repetitions of each stimulus in the phonetic acoustic space) and 10 prosodic blocks (490 trials total, 10 repetitions of each stimulus in the prosodic acoustic space). Blocks alternated between phonetic and prosodic tasks, with short breaks interspersed. The order of trials in each block was randomized. The entire experiment lasted approximately 60 min. Participants completed the experiment online, at home in a quiet room alone, using either headphones (five amusics, seven controls) or external speakers (six amusics, four controls). Two participants did not have computers with sufficient sound capability and were therefore tested in a laboratory at Birkbeck College. For all participants, testing was conducted via the Gorilla web experiment platform (www.gorilla.sc/about; Anwyl-Irvine, Massonnié, Flitton, Kirkham, & Evershed, 2019).

#### Statistical analysis

Data were analyzed with R (R Core Team, 2018). Cue weights were calculated by constructing a multiple logistic regression for each participant (separately for phonetic and prosodic paradigms) with F0 and Duration as factors (on integer scales from 1–5 for the phonetic task and 1–7 for the prosodic task, according to the number of stimulus increments). The coefficients estimated from these models were then normalized such that the F0 and Duration weights summed to one to indicate the relative perceptual weight of each acoustic dimension in categorization responses (Holt & Lotto, 2006; Idemaru et al., 2012). A large coefficient for F0 relative to Duration indicated that the F0 factor explained more variance in participants’ categorization judgments than the Duration factor. To test for group effects, the Cue Weights for F0 and Duration were extracted for each participant and subjected to an independent-samples *t* test, separately for phonetic and prosodic tasks. Because the distributions of pitch thresholds were non-normal, the relationships between pitch and duration thresholds and cue weights were tested with Kendall’s τ-b.

### Results

#### Pitch, duration, and speech-in-noise thresholds

**Figure 2 fig2:**
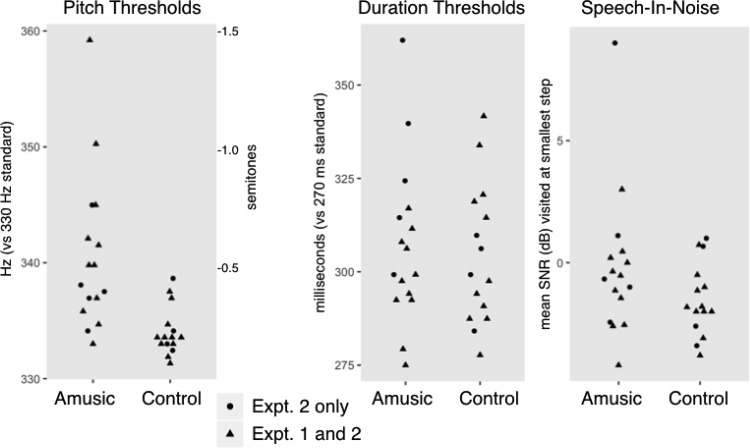
Thresholds for detecting pitch changes, duration changes, and for speech-in-noise.

Among the subset of participants who took part in Experiment 1, amusic participants, compared with controls, had significantly higher pitch thresholds (Wilcoxon’s Rank Sum W = 107, *p* = .002), but similar duration (W = 50, *p* = .51) and speech-in-noise thresholds (W = 78.5, *p* = .25). The pattern held across all participants: amusics as a group had higher pitch thresholds than controls (W = 29, *p* < .001), but did not differ from controls in tone duration discrimination (W = 129, *p* = .74) or speech-in-noise threshold (W = 155.5, *p* = .17; Figure 2). For the amusic group, all pitch discrimination thresholds were below 1.5 semitones, assuring that our manipulation of the F0 dimension in two-semitone steps in the cue weighting paradigms would be perceptible even to amusic participants.[Fig-anchor fig2]

#### Phonetic cue weighting

**Figure 3 fig3:**
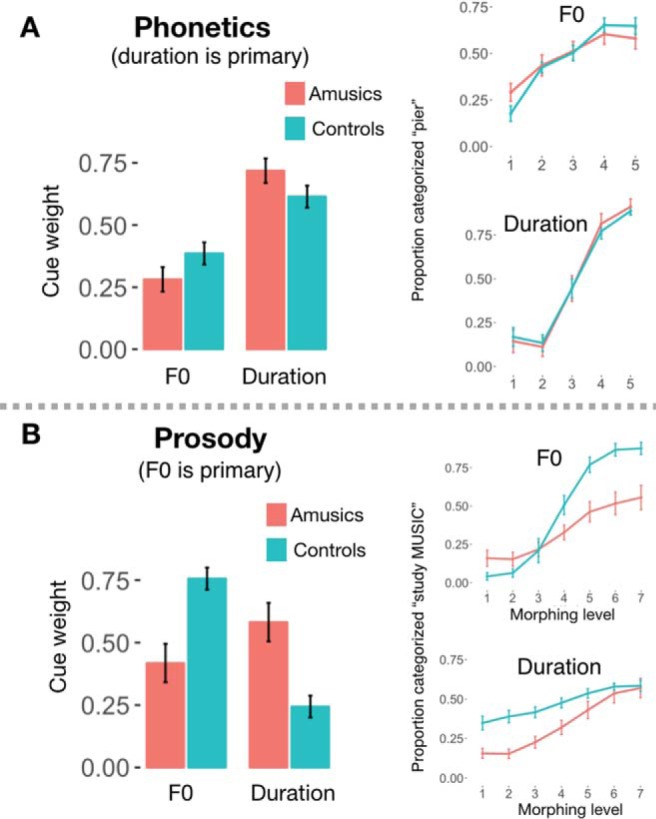
Comparison of F0 and duration cue weights for phonetic and prosodic perception. (A) Phonetic Cue Weighting. Mean perceptual weights plotted by group and condition (left). Mean categorization response plotted at each level of F0, collapsed over duration; and each level of duration collapsed over F0. (B) Prosodic cue weighting. Analogous plots for the prosodic categorization. Bars indicate standard error of the mean.

**Figure 4 fig4:**
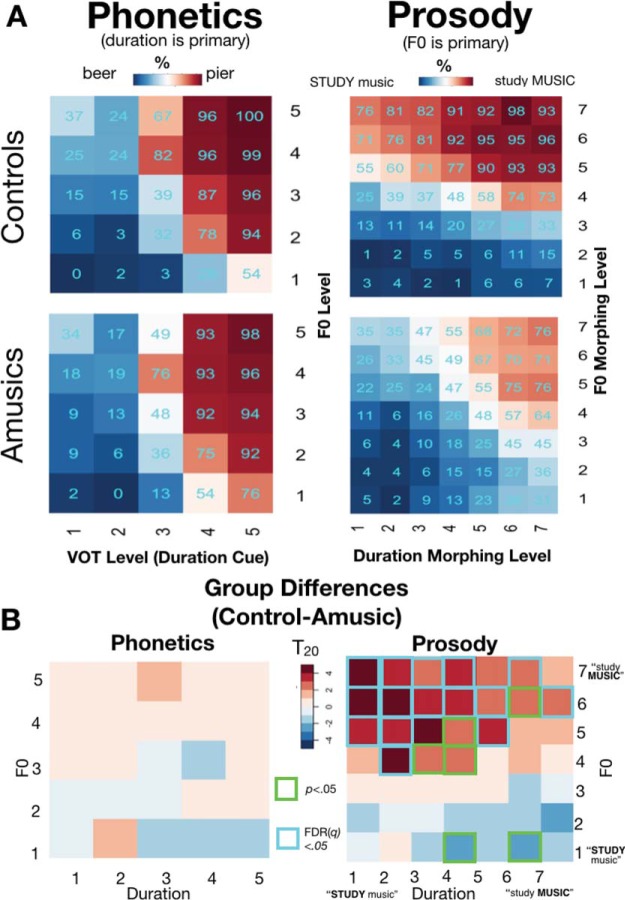
When F0 and duration cues conflict, amusics rely on duration when categorizing prosody. (A) Heatmaps indicate proportion of trials categorized as *study MUSIC* (for the prosody portion) or *pier* (phonetics portion), for the Control and Amusic groups. (B) Group difference (Control—Amusic) heatmaps displaying *t* statistics. When duration and F0 conflicted in the prosody task (duration indicated emphasis on STUDY, but F0 indicated emphasis on MUSIC; upper-left quadrant of stimulus space), amusic participants chose the duration-based response more often than controls. Teal outlines indicate significant group differences (corrected for multiple comparisons). Uncorrected results (*p* < .05) are indicated with green outlines.

The normalized cue weights for the amusic and control groups were compared with *t* tests because normalizing the F0 and duration cue weights relative to each other (so they sum to 1) causes them to be nonindependent, and therefore a Group × CueType interaction test was inappropriate. Recall that duration (VOT) was expected to be the primary cue for the phonetic task. Accordingly, the magnitude of the pitch and duration weights did not differ between groups (Figure 3A; group effects *t*_[20]_ = 1.58, *p* = .13). Although the mean responses between amusics and controls (Figure 4B) did not differ, the results of the independent-samples *t* test of perceptual cue weights across groups across each of the 25 stimuli are presented in Figure 4B for illustrative comparison. Here, again, categorization performance across groups was similar for each stimulus in the acoustic space. No group differences were detected even at a very lenient (uncorrected for multiple comparisons) threshold of *p* < .05.[Fig-anchor fig3][Fig-anchor fig4]

**Figure 5 fig5:**
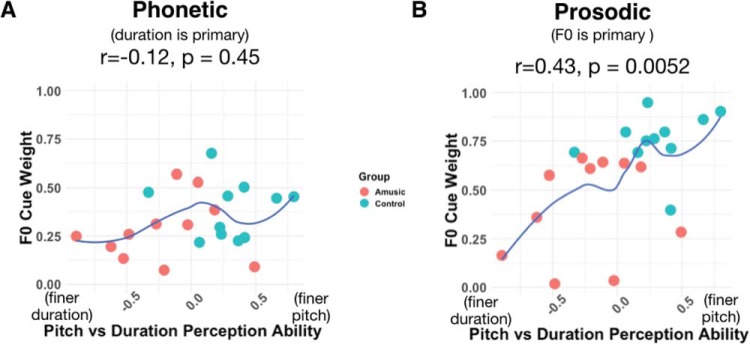
Correlation between perceptual discrimination and perceptual cue weight. The figures plot relative pitch versus duration thresholds as a function of the relative perceptual weight of F0 in phonetic (A) and prosodic (B) judgments. The correlation shown is Kendall’s tau-b. Loess curves are plotted to indicate direction of trends. (A) In the phonetic task, for which F0 is a secondary perceptual dimension, there was no significant correlation between perceptual thresholds and normalized perceptual weight of F0. (B) In the prosodic task, for which F0 is the primary perceptual dimension, individuals with finer pitch discrimination thresholds tended to rely more on F0 in prosodic focus judgments, even though the F0 differences in the prosodic task were large enough to be perceptible by all participants.

Finally, we calculated a standard score reflecting participants’ relative ability to discriminate pitch and duration in complex tones based on their perceptual thresholds, and tested whether this metric was related to participants’ relative perceptual weightings in the phonetics task. To obtain a standard score, the standards were subtracted from each participant’s pitch and duration thresholds F0 (for pitch, 330 Hz) or standard duration (270 ms) used in the psychophysics test, then divided by the standard deviations of these distributions across participants. The standard scores for pitch and duration were then combined with an asymmetry ratio [(Duration − Pitch)/(Pitch + Duration)] such that higher values indicated finer pitch than duration thresholds and lower values indicated the reverse. The relative ability to discriminate tone pitch versus duration did not correlate with perceptual weight of F0 in phonetic categorization (Kendall’s τ-b *r* = −0.12, *p* = .45; Figure 5A).[Fig-anchor fig5]

#### Prosodic cue weighting

In the prosodic cue weighting task, for which F0 was the primary cue, the results were strikingly different. The relative F0 perceptual weights were higher in controls than amusics, whereas duration weights were higher for amusics than controls (both group comparisons *t*_[20]_ = 3.81, *p* = .001, Figure 3B). This suggests that amusics, who do not process pitch reliably in general, indeed perceptually weighted duration over F0. As mentioned above, an interaction test on the normalized (relative) cue weights is inappropriate because they sum to 1 (e.g., if a participant’s pitch weight were 0, his or her duration weight would necessarily be 1); however, we note that an ANOVA on the raw cue weights (before normalization) further confirmed this pattern (interaction of CueType [pitch vs. duration] × Group [Amusic vs. Control], *F*_[1,40]_ = 10.45, *p* = 0.002, η_p_^2^ = 0.21).

To provide a finer-grained understanding of each group’s weighting, Figure 4A plots mean responses for each group across each of the 49 stimuli depicted schematically in Figure 1. As in the phonetic cue weighting task, we compared these matrices cell-by-cell (two-sample *T* tests Controls > Amusic, FDR-corrected, Figure 4B). Unlike in the phonetic task for which Control and Amusic participants performed similarly, many group differences emerged in the prosodic categorization task, where controls relied relatively more on pitch versus duration compared with amusics. All (multiple-comparisons-corrected) significant differences were in the top half of the matrix, where F0 cued an emphasis on *MUSIC*. Most (12 of 16) of these significant group differences occurred in the 16 stimuli of the upper-left quadrant, where emphasis was placed on *STUDY* by duration cues, and *MUSIC* by F0 cues. In this ambiguous quadrant of the stimulus space, where F0 and duration signaled differing interpretations, amusics relied on the duration cues to make their response more often than did controls.

Across both the amusic and control groups, participants with finer pitch than duration discrimination thresholds tended to have higher F0 (than duration) cue weights (Kendall’s τ-b *r* = .43, *p* = .005; Figure 5B).

Finally, we confirmed statistically the greater group differences in perceptual weightings in the Prosody task compared with the Phonetic task (interaction of Group × Experiment predicting normalized F0 cue weights, *F*_[1,40]_ = 4.44, *p* = .04, η_p_^2^ = 0.10).

### Discussion

In two categorization tasks, participants with amusia and control participants categorized stimuli varying across F0 and duration dimensions according to a phonetic distinction and a prosodic distinction. The phonetic and prosodic categorization tasks varied in the extent to which category membership was cued by information from the F0 dimension versus the duration dimension. For the phonetic distinction, duration had been established by prior literature to be the primary dimension (Haggard et al., 1970; Massaro & Cohen, 1976, 1977), whereas for the prosodic distinction, F0 was known to be primary (Chrabaszcz et al., 2014). We found that when duration was the primary dimension (in the phonetics task), the amusic and control groups categorized similarly, each relying predominantly on duration (VOT) to signal category membership. However, when F0 was primary (in the prosody task), classification behavior differed between the groups: controls classified ambiguous stimuli (for the most part) according to the primary dimension (F0), as predicted by prior research (Breen et al., 2010) whereas amusics tended to classify the very same stimuli instead according to duration. Crucially, this difference could not be explained by the amusic participants being simply unable to hear the F0 cues, because (a) sensitivity to the F0 dimension was observed in both the phonetic and prosodic task and (b) the differences in F0 across stimulus levels in each task were large enough to be above-threshold and clearly perceptible to the amusics. Indeed, they were well above amusics’ independently measured pitch thresholds.

Many prior studies have reported large individual differences in dimensional weighting during speech perception across a variety of different contexts, including Mandarin tone perception (Chandrasekaran et al., 2010), place of articulation (Hazan & Rosen, 1991), stop consonant length (Idemaru et al., 2012), vowel contrasts (Kim et al., 2018), and voicing (Schertz, Cho, Lotto, & Warner, 2015; Kong & Edwards, 2016). Here, we provide evidence that individual differences in dimensional weighting do not solely reflect measurement noise, but instead can indicate stable listening strategies: amusic individuals focus on the dimensions which they are best able to process and down-weight the dimensions which they have historically found less useful. This finding suggests, more generally, that differences in dimensional weighting may reflect weighting of perceptual evidence by precision, that is, inverse variance (Feldman & Friston, 2010). In other words, an ideal strategy for one listener may be suboptimal for another because of individual differences in the precision of auditory perception. Thus, we suggest that the computational problem which must be solved during speech perception differs across listeners, because each listener experiences a unique set of constraints.

Amusics’ adoption of this specialized perceptual strategy could help explain why amusics do not generally report difficulty with speech in everyday life (Liu et al., 2010), despite displaying deficits when tested on speech perception tasks requiring the use of F0 information. Indeed, we further found that psychophysical thresholds across all participants, controls and amusics, correlated with the relative weighting of F0 versus durational information in the prosodic task, confirming a link between the precision of perception along a given primary dimension and the extent to which listeners weight that dimension in perceptual categorization. This relationship between perceptual acuity and categorization strategies could extend to other perceptual domains as well, such as identification of characteristics of environmental sounds (Lutfi & Liu, 2007).

Amusic and control groups overlapped in pitch discrimination thresholds, which may initially seem surprising, given that the amusic participants were defined as having severe difficulties with pitch perception. However, amusia is not defined solely on the basis of poor pitch discrimination, but on the basis of performance on batteries of music perception, specifically on tests of comparing melodic sequences. Performance on melodic sequence tasks can be driven by difficulties with low-level encoding of pitch, but could also be driven by difficulties with other aspects of pitch processing, including pitch memory, perception of higher-level pitch structures such as tonal relationships, and so forth. In this article, because of testing time constraints, we evaluated pitch versus duration perception abilities using relatively simple psychophysical tests rather than comprehensive batteries. In practice, however, we suggest that cue weighting is fundamentally driven by a range of pitch-related and duration-related skills, which include but are not limited to low-level encoding and discrimination.

The results of Experiment 1 revealed that amusics weight F0 cues relatively less than duration cues, compared with controls. This was true for the prosodic task where F0 was typically the primary dimension, but not true for the phonetic task, where duration is typically primary. This suggests that listeners will only change cue weighting in response to perceptual difficulties if the dimension they have difficulty processing is the primary cue for a given linguistic feature. An alternative explanation, however, is that these findings may reflect a specific amusic deficit for integration of pitch information across longer time frames, rather than a simple encoding deficit. Indeed, in the prosody paradigm, F0 information unfolded over time in the order of seconds, whereas in the phonetic paradigm the F0 excursion was over milliseconds. Future work could distinguish between these two explanations (primary vs. secondary cue; long vs. short time scale) by examining cue weighting in amusics and controls for perception of a prosodic feature for which duration is a dominant cue, such as lexical stress (Mattys, 2000). Overall, these results indicate that the manner in which auditory dimensions map to linguistically relevant categories differs across listeners, and that these individual differences in perceptual weighting reflect, at least in part, variability in how well listeners can perceive auditory dimensions. A particular dimension, for example, may be primary for one listener but secondary for another, leading to differing categorization strategies for the same stimuli across different listeners.

## Experiment 2

Whereas Experiment 1 focused only on speech perception, redundancy in acoustic signals ought to aid with perception of music as well. In Experiment 2 we examined whether cue redundancy in more naturalistic stimuli from speech, as well as music, leads to robustness in the face of perceptual deficits. In other words, we tested whether the perceptual strategy employed by amusics in Experiment 1—where amusics rely less on pitch information when it is a primary cue, focusing instead on other sources of information about speech—can be successful enough to lead to perceptual categorization performance equivalent to that of control participants.

Although amusia is canonically a problem with using pitch information to perceive music, there are temporal cues to certain musical structures (such as the beginnings and ends of phrases; Palmer & Krumhansl, 1987) that could theoretically enable amusics to compensate for poor pitch perception and successfully track musical features. To examine this possibility, first, in a music perception test, participants categorized phrases as complete or incomplete when they could rely on pitch alone, duration alone, or both dimensions simultaneously. If so, we predict that amusics should be able to detect musical phrases when durational cues are present, but not when only pitch cues are present.

Second, we investigated whether temporal dimensions in speech provide sufficient information for amusics to compensate for poor pitch perception, enabling categorization of prosodic information at a level similar to that of controls. We tested this with two linguistic prosody tasks, one for which pitch/F0 is generally the main distinguishing cue and one where durational cues are primary. In both tasks we measured the extent to which participants could use F0 and duration dimensions to make categorical decisions about whether words had linguistic focus (or emphasis; e.g., “Mary likes to READ books, but she doesn’t like to WRITE them.”) or whether phrase boundaries were present or absent (“After John runs [phrase boundary], the race is over”). To do this, we manipulated stimuli such that participants needed to rely on the F0 dimension alone, the duration dimension alone, or could use both. Across both speech and music perception, we predict that if there is sufficient information in durational patterns for the extraction of structural features—that is, if pitch and duration are fully redundant—then amusics should perform similarly to controls in both the duration-only and combined F0 and duration conditions for both music and speech perception, and that amusics’ performance should be improved by the presence of redundant cues.

### Method

#### Participants

All 31 recruited participants took part in Experiment 2. For description of participants, see Experiment 1.

#### Procedure

This experiment was performed in a quiet laboratory at Birkbeck using headphones. The Linguistic Phrase Test was run first (∼20 min), followed by the Musical Phrase Test (∼20 min), then the Focus Test (∼30 min).

#### Musical phrase perception test

##### Stimuli

The stimuli consisted of 150 musical phrases taken from a corpus of folk songs (Schaffrath & Park, 1995). Musical phrases were synthesized as a sequence of six harmonic complex tones with 10-ms cosine onset and offset ramps. Stimuli were created in three conditions that manipulated the acoustic dimensions available to listeners. In the Combined condition, the musical phrase contained both pitch and duration information (as typical naturalistic melodies do). Fifty stimuli were selected from the database for this condition only, and 50 additional stimuli were selected for each of two additional conditions. In the Pitch condition, the pitch of the melody was preserved (as in the original version) but the durations were set to be isochronous and equal to the mean duration of the notes in the original melody. This is because although isochronous can be perceived as rhythmic (Brochard, Abecasis, Potter, Ragot, & Drake, 2003), isochronous durations do not provide durational cues to musical phrase boundaries. In the Duration condition, the original note durations were preserved but the pitch of the notes was made to be monotone at a pitch of 220 Hz. In an additional manipulation, half of the stimuli presented in each condition formed a complete musical phrase with the notes in an unmodified sequential order—these could be perceived as a *Complete* musical phrase. The other half were made to sound *Incomplete* by presenting a concatenation of the second half of the musical phase and the first half of the next musical phrase in the song. The order of the notes within the two halves was preserved. Thus, the resulting *Incomplete* stimuli contained a musical phrase boundary that occurred in the middle of the sequence, rather than at the end.

##### Procedure

**Figure 6 fig6:**
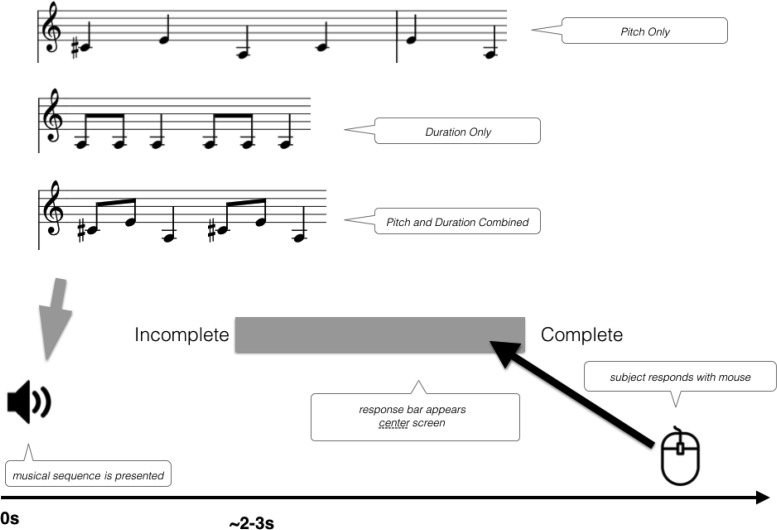
Schematic of trial structure for the Musical Phrase Test. Participants heard either a complete musical phrase or a musical sequence that straddled the boundary in between two musical phrases. They then indicated how complete they thought the phrase sounded by clicking with a mouse at a point along a response bar.

On each trial, a musical note sequence was presented to the participant through headphones. After the sound finished playing, a response bar appeared on the screen which was approximately 10 cm in width. Participants were tasked with deciding how complete each musical phrase sounded by clicking with their mouse on the response bar. (A continuous measure of completeness was used to avoid potential ceiling/floor effects attributable to bias on the part of individual listeners to hear phrases as complete or incomplete.) The word *Incomplete* was shown on the left side of the response bar, and the word *Complete* was shown on the right. Participants could click anywhere within the bar to indicate how complete they thought the phrase had sounded (see Figure 6). The next stimulus was played immediately after a response. Participants judged three blocks of 50 trials each, with a short break in between. As the study was aimed at understanding individual differences, the block order was always the same, with all the trials in a condition presented in a single block, in the same order (Combined Cues, then Duration Only, then Pitch Only). Within a block, half of the trials were Complete, and half Incomplete. The main outcome measure was the raw rating difference between Complete and Incomplete trials for each condition. Trials were blocked by condition because the three conditions for the Musical Phrase test were saliently different, and so fully randomizing trials could have led to substantial task-switching costs on a trial-wise basis.[Fig-anchor fig6]

#### Linguistic focus perception task

##### Stimuli

**Figure 7 fig7:**
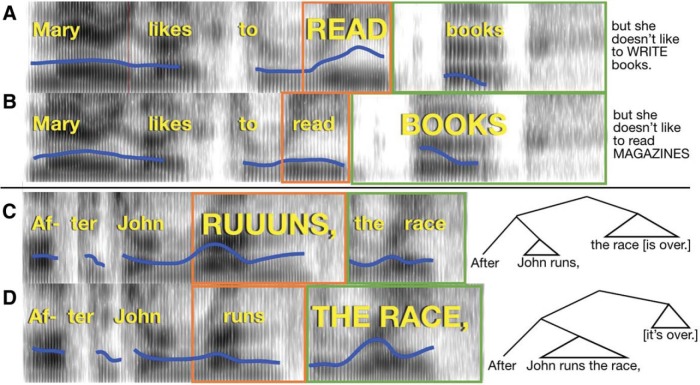
Pitch and duration correlates of emphatic accents and phrase boundaries. Spectrograms of stimuli used in the experiment (time on horizontal axis, frequency on vertical axis, and amplitude in grayscale), with linguistic features cued simultaneously by F0 and duration (the “Combined” condition). Blue line indicates F0 contour. Width of orange and green boxes indicate duration of the words within the box. (A) Emphatic accent places focus on *read*. Completion of the sentence appears to the right. (B) Emphatic accent places focus on *books*; sentence completion is at right. (C) A phrase boundary occurs after *runs.* (D) A phrase boundary occurs after *race*. Syntactic trees are indicated at right to illustrate the structure conveyed by acoustics of the stimuli.

The stimuli consisted of 47 compound sentences with an intervening conjunction created specifically for this study. Each of the sentences was read with emphasis to create had two versions: *early focus*, where emphasis occurred early in the sentence and served to contrast with a similar word later in the sentence (e.g., “*Mary likes to READ books*, but she doesn’t like to WRITE them,” focus indicated by upper-case letters), and *late focus*, where the focus occurred slightly later in the sentence (e.g., “*Mary likes to read BOOKS*, but she doesn’t like to read MAGAZINES,” focus indicated by upper-case letters; see Figure 7A and 7B and online supplemental materials Section 1).[Fig-anchor fig7]

We recorded these sentences (44.1 kHz, 32-bit) using a Rode NT1-A condenser microphone as they were spoken by an actor who placed contrastive accents to emphasize the capitalized words. Recordings of both versions of the sentence were obtained and cropped to the identical portions (underlined above). We used the standard STRAIGHT (Kawahara & Irino, 2005) morphing pipeline, which involves decomposing the signal of the two recordings into three parts: first, the F0 structure was extracted from voiced segments of each of the two utterances; next, aperiodic aspects of the signal (such as obstruents) were identified and analyzed; then, the filter characteristics of the signal (similar to the power spectrum) were calculated. Finally, the two morphing substrates (speech from each recording decomposed into these component parts) were manually time aligned by marking corresponding anchor points in both recordings, such as the occurrence of salient phonemes, so that morphs reflect the temporal characteristics of the two initial recordings, to varying degrees. Once time-aligned, morphs can be created by varying the degree to which each morphing substrate contributes information about F0, aperiodicity, filter, and duration. The relative contribution of each morphing substrate is given as the *morphing level*, on a scale from 0% to 100%, with 50% indicating equal contribution from each of the two individual recordings. For our study, we only manipulated the F0 and durational characteristics of the morphs, and left the other aspects set to the midpoint, such that each recording contributed equally. We then produced six different kinds of morphs by varying the amount of pitch-related (F0) and duration information either independently or simultaneously. For *F0 only* stimuli pairs, the late and early focus sentences differed only in F0 excursions. The temporal morphing level between the two versions was held at 50% while the F0 was set to include 75% of the early focus version or 75% of the late focus version recording. This resulted in two new recordings that differed in F0 excursions, but were otherwise identical in terms of duration, amplitude and spectral quality. For *duration only* stimuli, we created two more morphs that held the F0 morphing proportion at 50% while the duration proportion was set to either 75% early focus or 75% late focus. The output differed only in duration, and were identical in terms of F0, amplitude and spectral quality. Finally, we made *naturalistic* stimuli where both F0 and duration information contained 75% of one morph or the other, and thus F0 and duration simultaneously cued either an early or late focus reading. Across pairs of stimuli, the mean largest difference in F0 between early and late focus was 8.1 semitones. All files were saved and subsequently presented at a sampling rate 44.1 kHz with 16-bit quantization.

##### Procedure

**Figure 8 fig8:**
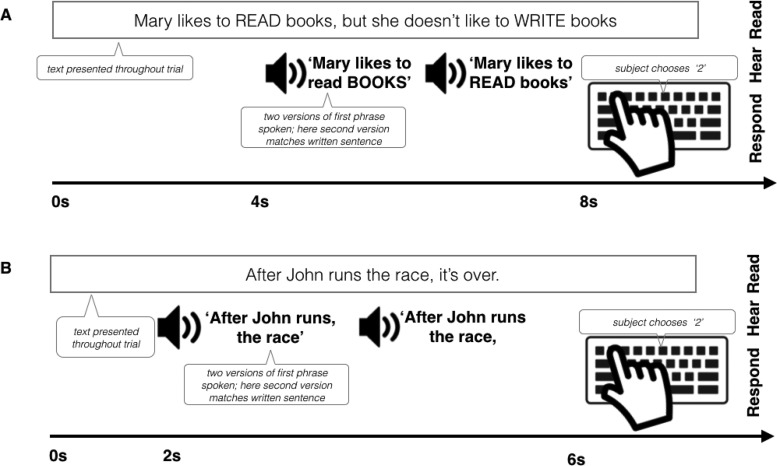
Example trial structure for the linguistic focus test (A) and the linguistic phrase test (B). First, a single sentence was presented visually, and the participants read it to themselves. Next, two auditory versions of the first part of the sentence were played sequentially, only one of which matched the focus pattern of the visually presented sentence. Participants then indicated which auditory version matched the onscreen version with a button press.

Stimuli were presented with Psychtoolbox in Matlab. Participants saw sentences presented visually on the screen one at a time, which were either early or late focus (see paradigm schematic in Figures 7A, 7B, and 8A). The emphasized words appeared in all upper-case letters, as in the examples above. Participants had 4 s to read the sentence to themselves silently and imagine how it should sound if someone spoke it aloud. Following this, participants heard the first part of the sentence spoken aloud in two different ways, one that cued an early focus reading and another that cued late focus. Participants were instructed to listen and decide which of the two readings contained emphasis placed on the same word as in the text sentence. After the recordings finished, participants responded by pressing “1” or “2” on the keyboard to indicate whether they thought the first version or second version was spoken in a way that better matched the on-screen version of the sentence. The correct choice was cued either by F0 or duration exclusively, or both together. The serial order of the sound file presentation was randomized (uniquely for each participant). The stimuli were divided into three lists (47 trials each) and counterbalanced such that each stimulus appeared once in each condition (F0, duration, and both). For 23 of the stimuli, two of the presentations were early focus, and one was late focus; for the remaining stimuli, two presentations were late focus and one was early. The trials were presented in the same order for every participant. The entire task lasted approximately 30 min.[Fig-anchor fig8]

#### Linguistic phrase perception test

##### Stimuli

The stimuli consisted of 42 short sentences with a subordinate clause appearing before a main clause (see Figure 7C and 7D). About half of these came from a published study (Kjelgaard & Speer, 1999) and the rest were created for this test (see the online supplemental materials, Section 2). The sentences appeared in two conditions: an *early closure* condition, for which the subordinate clause’s verb was used intransitively, and the following noun was the subject of a new clause (“After John runs, the race is over”); and *late closure*, where the verb was transitive and took the following noun as its object, causing the phrase boundary to occur slightly later in the sentence (“After John runs the race, it’s over”). Both versions of the sentence were lexically identical from the start of the sentence until the end of the second noun.

A native Standard Southern British English-speaking male (trained as an actor; the same voice model as for the focus task) recorded early and late closure versions of the sentences in his own standard Southern English dialect. The recordings were cropped such that only the lexically identical portions of the two versions remained, and silent pauses after phrase breaks were excised. The same morphing proportions were used as in the focus task—with early or late closure cued by 75% morphs biased with pitch, duration or both combined. As was done with the linguistic focus task, the stimuli were crossed with condition (F0, duration, and both) and early versus late closure and divided into three counterbalanced lists. Across pairs of stimuli, the mean largest difference in F0 between early and late closure was 6.4 semitones.

##### Procedure

The procedure for the Linguistic Phrase test was similar to that of the Linguistic Focus Test. Participants saw sentences presented visually on the screen one at a time, which were either early or late closure, as indicated by the grammar of the sentence and a comma placed after the first clause (Figure 8B). They then had two seconds to read the sentence to themselves silently and imagine how it should sound if someone spoke it aloud. Following this period, participants heard the first part of the sentence (which was identical in the early and late closure versions) spoken aloud, in two different ways, one that cued an early closure reading and another that cued late closure. The grammatical difference between the two spoken utterances on each trial was cued by either F0 differences, duration differences, or both F0 and duration differences. Participants completed three blocks of 42 trials. Stimuli were counterbalanced such that each stimulus appeared once in each condition, and half the of presentations were early close and half were late close. The trials were presented in the same order for every participant. The task was performed in a lab at Birkbeck and lasted approximately 25 min.

#### Psychophysics

As described in Experiment 1, thresholds for pitch and duration discrimination, and ability to hear speech in noise, were measured using the MLP toolbox (Grassi & Soranzo, 2009).

#### Statistical analysis

Data were analyzed with R. For the musical phrase, linguistic focus and linguistic phrase tests, linear mixed effects models were estimated using *lme4*, with Group (Amusic or Control), Condition (Pitch, Duration or Combined), and their interaction entered as fixed effects, and Item and Participant as random intercepts. *p* values for these effects were calculated with likelihood ratio tests of the full model against a null model without the variable in question. Comparisons of predicted marginal means were performed with *lsmeans.*

The dependent variable for the Musical Phrase Test was calculated by identifying the raw response value between −50 and 50 (for each trial) based on the position along the response bar on which the participant clicked, with −50 corresponding to responses on the extreme end of the Incomplete side of the scale. The sign of the data point for Incomplete trials was then inverted so that more positive scores always indicated correct performance and greater scores indicated more accurate categorization of musical phrases.

The dependent variable that was entered into the model for the Focus and Linguistic Phrase tests was whether each response was CORRECT or INCORRECT. Because the dependent variable was binary, we used the generalized linear mixed models (*glmm*) function in the *lmer* package to estimate mixed effects logistic regressions, and we report odds ratios as a measure of effect size.

### Results

#### Musical phrase perception

To recap, the musical phrase perception test (see Figure 6) tested participants’ ability to perceive the extent to which a series of notes resembled a complete musical phrase, with acoustic dimensions of F0, duration, or their combination available to support the judgment. The availability of acoustic dimensions affected participants’ accuracy in identifying complete versus incomplete phrases. Accuracy was highest when both cues were present, lowest when only the F0 dimension was present, and intermediate when only the duration dimension was present (main effect of Condition χ^2^[4] = 15.6, *p* = .004, see Table S1 in the online supplemental materials for pairwise statistics). Compared with controls, amusics were overall less accurate (main effect of Group χ^2^[3] = 10.25, *p* = .017) and also differentially affected by which dimensions were present (Group × Condition interaction χ^2^[2] = 8.6, *p* = .013). FDR-corrected pairwise tests showed that when only the pitch-relevant F0 dimension was available, the average amusic’s performance was significantly lower than controls, *t*(119.93) = 2.86, *p* = .022 (Table S1 in the online supplemental materials). By contrast, when amusics could rely on duration alone, or both pitch and duration together (as in the Combined condition, where cues were present as in naturalistic melodies), amusics and controls did not differ significantly.

#### Linguistic focus test

**Figure 9 fig9:**
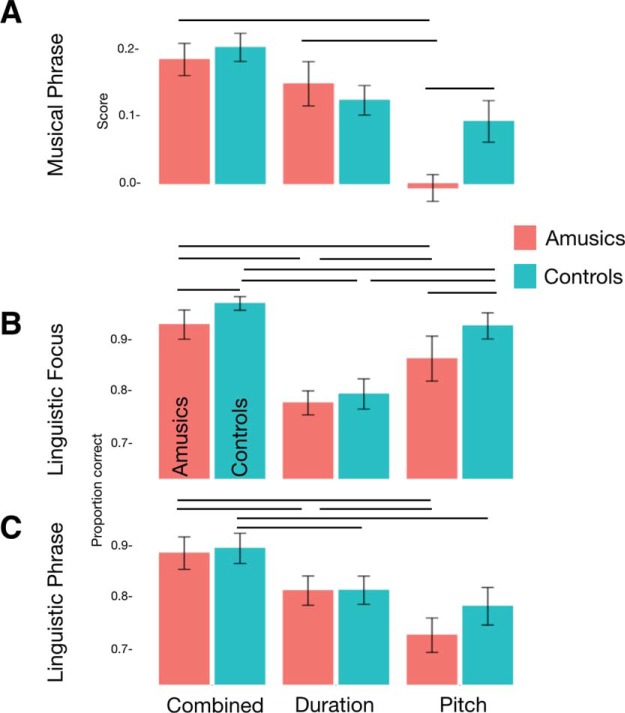
Results of the Linguistic Focus, Linguistic Phrase, and Musical Phrase tests. Error bars show standard error of the mean and horizontal lines indicate significant pairwise contrasts (FDR-corrected) mentioned in results.

The linguistic focus test (schematic Figure 8A) measured participants’ ability to detect where a contrastive accent was placed in a sentence, based on only one type of acoustic dimension (F0 or Duration) or both combined (as in natural speech). As shown in Figure 9B and Table S2 in the online supplemental materials, overall both groups performed best when they heard F0 and duration cues together, worst when only duration cues were present, and in between when there were only F0 cues (main effect of Condition χ^2^[4] = 168.4, *p* < .001). This suggests that both groups benefited from redundant information, and that F0 was more useful for detecting focus than duration, in line with results from Experiment 1. On the whole, controls performed more accurately than amusics (main effect of Group χ^2^[3] = 14.63, *p* = 0.002). However, the two groups were differentially affected by whether F0 or duration cues were present in the stimuli (interaction of Group × Condition χ^2^[2] = 12.05, *p* = .002). When relying on duration alone, amusics performed similarly to controls, but when they needed to rely on F0 they performed significantly less accurately (odds ratio = 2.00, *z* = 2.39, *p* = .019; Table S2 in the online supplemental materials). This disadvantage held where F0 was the sole cue, as well as in the Combined F0 + duration cue condition. This result stands in contrast to that in the musical phrase test, where performance in the combined (pitch + duration) condition did not differ between groups.[Fig-anchor fig9]

#### Linguistic phrase test

The Linguistic Phrase Perception Test (schematic Figure 8B) measured participants’ ability to detect phrase boundaries in speech which are cued by F0 only, duration only, or both F0 and duration. Cue type affected performance across groups (main effect of Condition χ^2^[4] = 83.06, *p* < .001). Participants performed least accurately when they had to rely on F0 cues alone, better when they relied on duration alone, and most accurately when both F0 and duration were present together (see Figure 9C and Table S3 in the online supplemental materials). This is consistent with prior evidence suggesting that duration is a more reliable cue for the detection of phrase boundaries than pitch (Streeter, 1978). As in the Focus test, redundant cues benefitted both groups, but in contrast to the pattern in the Focus Test, duration was a more reliable cue to linguistic phrase boundary perception than pitch.

Amusics did not differ significantly from controls in overall accuracy (main effect of Group χ^2^[3] = 2.69, *p* = 0.44) nor was the groups’ performance significantly differently affected by which acoustic cues were present (interaction of Group × Condition χ^2^[2] = 2.33, *p* = .31). Because we had hypothesized a priori that amusics would rely more on duration than F0 (and that controls would show similar performance across the two conditions), we conducted pairwise contrasts to test this prediction. Amusics did indeed show significantly greater accuracy with duration than with F0 cues (odds ratio = 1.63, *z* = 3.56, *p* = .001; Table S3 in the online supplemental materials), whereas controls did not. (For completeness, all other [post hoc] pairwise comparisons are also reported).

#### Correlations between psychophysical thresholds and musical phrase, linguistic phrase, and linguistic focus tests

We examined the relationship between basic auditory processing and the perception of prosody and music by testing whether individuals’ thresholds for detection of pitch change and duration change were correlated with performance scores on any of the conditions of our three main tasks. Pitch psychophysics thresholds were correlated with performance on the Focus Test when both pitch and duration cues were present (*r*_t_ = −0.31, *p* = .026) or when pitch alone was a cue (*r*_t_ = −0.3, *p* = .029). Pitch thresholds were also correlated with Musical Phrase Test performance when pitch cues were the sole cue to phrase endings (*r*_t_ = −0.29, *p* = .023). No significant correlations emerged for duration or speech in noise. Scatter plots are shown in the Figures S1–S3 in the online supplemental materials.

### Discussion

Music and speech carry information across multiple acoustic dimensions, with distinct dimensions often providing redundant information to support perceptual decisions. We tested whether this multidimensional redundancy makes music and speech robust to individual differences in perceptual abilities. We found that amusics perceived both speech and music well when they could rely on a redundant, unimpaired channel (duration), either on its own, or together with F0 or pitch. Furthermore, for musical and linguistic phrase perception, amusics were able to achieve equivalent performance to control participants when boundaries were redundantly cued by F0/pitch and duration. Thus, redundancy in the production of communicative signals makes possible robust message transmission in the face of individual differences in auditory perception. Indeed, in the two linguistic tasks, performance in the Combined cue condition was greater than either of the single cue conditions, suggesting that typically developed individuals also benefit from speech’s built-in redundancies.

Musical aptitude is often measured with tests that target specific domains like perception of melody or rhythm (Gordon, 2002; Wallentin, Nielsen, Friis-Olivarius, Vuust, & Vuust, 2010). This is, however, unlike actual music listening in the real world. Naturally produced musical structures, such as musical phrases, are often conveyed by simultaneous (i.e., redundant) cues (Palmer & Krumhansl, 1987). Here, we find no evidence that amusics’ intuitions about musical phrase structure are impaired as long as durational cues are present. Although amusics may not fully appreciate aspects of music that relate to pitch, we show that they can parse musical structures when another relevant cue is available. Future work should investigate whether this spared musical perception in amusics extends to other musical features which are communicated by redundant cues, such as musical beat perception (Hannon, Snyder, Eerola, & Krumhansl, 2004).

The literature has shown inconsistent findings regarding whether pitch and duration are perceived independently or interactively during music perception and cognition. Palmer and Krumhansl (1987), for example, found that duration and pitch cues carry equal weights in musical phrase perception, without additional benefits from being able to combine the two, while Prince (2014) found that rhythm and melodic contour were independent predictors of melodic similarity judgments, with no interactions between the two. On the other hand, Boltz, 1989a and 1989b found an interaction between temporal accent and effects of tonality on perception of resolution at the end of melodies, whereas Prince (2011) and Prince and Loo (2017) found asymmetric interactions between pitch and time: pitch characteristics influenced temporal goodness judgments and expectancy ratings, but temporal characteristics did not influence pitch goodness judgments and expectancy ratings. Our findings support a mixture of independent and interactive accounts of pitch and time processing in music perception. In our control participants, we found that performance was similar in single-cue and dual-cue conditions, whereas the amusic participants demonstrated a gain from redundancy. Thus, the extent to which these two dimensions are processed interactively may depend on participants’ skill at processing each dimension, which may, in turn, help determine the dimensions’ relative salience. For example, we would predict that influence of pitch characteristics on temporal ratings would be lessened or nonexistent in amusics.

One possible outcome was that amusics would show superior duration processing that they had developed to compensate for their pitch deficit. The data here do not support this. The amusics showed similar—and not significantly more accurate—duration perception ability compared with controls across the music and language tests, as well as similar psychophysical duration discrimination thresholds. The present data suggest that rather than developing exceptional duration processing ability, all that may be necessary to achieve normal speech perception is a reweighting in perception to emphasize dimensions for which perception is more accurate.

We have primarily interpreted group differences in performance on these musical and prosodic categorization tests as reflecting relative weighting of different perceptual dimensions. Another possible interpretation of our results, however, is that they reflect differences in participants’ internal models of how phrase boundaries and linguistic focus should be produced. That is, upon reading the visual stimulus, participants may have been internally generating an estimate of what that stimulus would sound like, and then comparing that motor/auditory simulation to the subsequent auditory input. On the other hand, inasmuch as speech perception in general may always involve a component of motor imagery (i.e., the motor theory of speech, Liberman & Mattingly, 1985), these perceptual and internal motor model interpretations may not be as different as they initially appear. Moreover, reports of preserved pitch production in amusia (Loui, Guenther, Mathys, & Schlaug, 2008) argue against an account of our results which primarily references group differences in models of motor production of prosodic features.

## General Discussion

There is a remarkable diversity in the use and weighting of perceptual dimensions in speech and music in the typical population (Chandrasekaran et al., 2010; Hazan & Rosen, 1991; Idemaru et al., 2012; Kim et al., 2018; Kong & Edwards, 2016; Schertz et al., 2015). How do such stable individual differences arise, and how do communication systems facilitate robust information transmission despite this variability? We hypothesized that when two perceptual dimensions convey the same communicative content, the relative ease with which an individual listener can extract information from those dimensions would bias the relative weighting of these dimensions. We tested this with an experiment of nature—people with congenital difficulties in processing pitch, but not duration—and asked whether such a specific perceptual starting state might bias these listeners away from using their unreliable dimension (pitch) and toward another that is processed more reliably (duration cues) during perceptual categorization. We also asked whether such biases would be specific to cases in which the unreliable dimension (pitch) tends to be the primary cue—for instance in prosodic emphasis—or if it would extend to cases in which it was a secondary cue (e.g., categorization of onset syllable voicing). Finally, we examined whether the existence of redundant cues in speech and music (i.e., the presence of both durational and pitch cues) makes possible relatively robust transmission of message-level information, despite profound individual differences in perceptual acuity.

We first asked the question of whether one’s relative ability to perceive certain auditory dimensions is indeed linked to perceptual weightings of those dimensions in speech perception. In the case of prosodic emphasis, typical listeners tend to rely mainly on F0, but people with pitch perception difficulties tended to rely less on F0. When F0 and duration cues conflicted, amusic participants generally categorized stimuli according to duration cues, whereas the control group tended to categorize the very same stimuli according to F0 cues. For onset syllable voicing (phonetic categorization, for which F0 is typically a secondary dimension), on the other hand, there were no significant differences between people with pitch perception difficulties and control participants. Importantly, the F0 frequency range used to convey information across both of these tasks was considerably greater than the pitch discrimination thresholds of each participant. Thus, although the participants with pitch perception difficulties could have used pitch perception for prosodic categorization, they may have learned over time that pitch cues conveyed by F0 contours were less useful for them compared with temporal/durational cues. In other words, reweighting away from use of F0 cues may reflect increased salience of information that is better perceived. These results, therefore, do not support additive models of perception, but are in accordance with the fuzzy logical model of perception (Massaro & Cohen, 1993), which suggests that unambiguous sources of information are up-weighted when other sources of information are ambiguous, and predictive coding theory (Feldman & Friston, 2010), which suggests that evidence is weighted in accordance with its precision (inverse variance).

Our results have implications for theoretical models of speech and music perception. In the past, theoretical models of speech perception have been constructed to model the behavior of ideal participants, approximated in practice by averages of group performance (Toscano & McMurray, 2010). Similarly, prior empirical investigations of how cues are integrated during speech and music perception have attempted to determine, across participants, which dimensions are weighted more strongly than others, with the implicit assumption being that dimensional weighting strategies primarily reflect the distribution of cues associated with the existence of certain phonetic, prosodic, and musical structures. For linguistic focus, for example, pitch has been described as the primary cue, while word duration has been described as a secondary cue providing supplementary information (Breen et al., 2010). Our data, on the other hand, suggest that dimensional weighting strategies reflect a combination of the statistical characteristics of the input and the unique combination of perceptual strengths and weaknesses possessed by an individual. In other words, even if an acoustic dimension such as pitch is statistically a more reliable cue to a linguistic or musical feature, nevertheless for some listeners the optimal strategy may not be to rely heavily on pitch cues—because those pitch cues must pass through the filter of an individual’s perceptual system, which may process pitch and duration more or less reliably than one another. As such, we suggest that theoretical models for how acoustic dimensions are integrated in speech and music perception as well as future empirical research on this topic should take into account perceptual fidelity at the individual level in addition to acoustical factors at the signal level.

Next, we asked whether the redundant information conveyed by pitch/F0 and duration is sufficient to enable reliable message transmission via prosody despite severe perceptual deficits. In a follow-up experiment, we found that although participants with pitch perception difficulties struggled to decode musical and prosodic information when conveyed only via spectral cues (pitch for music, F0 for voices), they performed equivalently to control participants when durational cues were added. Thus, the existence of redundant cues to structure in speech and music ensures that these systems are robust even to extreme perceptual deficits. Because of the redundancy built into speech and music, people with specific and even severe auditory deficits can perceive structure nonetheless.

To keep the experimental design simple, we only examined two auditory dimensions—pitch changes conveyed through F0, where we suspected our groups would show a difference, and duration, where we believed they would not. Outside the laboratory there are other cues that individuals could take advantage of, such as vowel quality, which is also associated with phrase boundaries, and pitch accents (Sluijter & van Heuven, 1996; Streeter, 1978). Accents also carry visual correlates, such as head movements, beat gestures, and eyebrow raises (e.g., Beskow, Granström, & House, 2006, Flecha-García, 2010; Krahmer & Swerts, 2007), which individuals may also be able to use to compensate for their pitch impairment in audiovisual speech perception. Moreover, top-down processes such as the use of lexical knowledge can also help disambiguate unclear speech (Connine & Clifton, 1987; Ganong, 1980) and talker identity cues from the visual modality help listeners to disambiguate acoustic-phonetic cues (Zhang & Holt, 2018). Individuals may be able to modify the extent to which they make use of any or all of these different sources of information in response to their idiosyncratic set of strengths and weaknesses. For example, individuals with widespread auditory processing problems may rely more heavily on top-down lexical information, or visual cues.

Our participants were all adults, and so could take advantage of decades of experience in perceiving speech. One open question is the length of time necessary for developing perceptual categorization strategies. Although correlations between auditory perception thresholds and speech perception are generally weak or nonexistent in adults listening to their native language, stronger correlations have been reported in young children (Bavin, Grayden, Scott, & Stefanakis, 2010; Boets, Wouters, van Wieringen, De Smedt, & Ghesquière, 2008) and adults in the initial stages of learning a foreign language (Chandrasekaran, Kraus, & Wong, 2012; Kachlicka, Saito, & Tierney, 2019), suggesting that robust perceptual strategies take time to develop. Auditory processing may, therefore, be a bottleneck for speech perception in the initial stages of acquiring a language, at which time listeners have not yet developed stable adult-like perceptual weights across acoustic dimensions (Idemaru & Holt, 2013; Mayo & Turk, 2004). In later stages of language acquisition, correlations between auditory processing and speech perception may dissipate due to increased perceptual redundancy as perceptual strategies mature, although language delays attributable to earlier problems with speech may persist (Rosen, 2003; Ramus, Marshall, Rosen, & van der Lely, 2013). This hypothesis could be tested by examining the relationship between cue weighting and auditory processing in children. Prior work has shown that compared with adults, children place more emphasis on primary cues to phonetic contrasts and less emphasis on secondary cues (Idemaru & Holt, 2013). If what makes a cue primary versus secondary is its relative salience, then children may initially focus on the cue they can best process, ignoring other sources of evidence.

That individuals can compensate for perceptual impairments through greater reliance on higher-fidelity auditory dimensions, along with evidence that perceptual weighting strategies can be altered through training (Francis et al., 2008; Lim & Holt, 2011), suggests that it may be possible to ameliorate speech perception deficits via the development of targeted auditory processing training batteries. In particular, participants could be trained to rely more heavily on the attributes which they can more easily track (e.g., participants with temporal perception deficits could be trained to focus on pitch-based cues to syntactic and phonological structure).

The extent to which individual differences in dimensional weighting are stable across tasks and individuals remains an open question. As discussed above, amusics rely less on F0 in prosodic but not phonetic categorization, suggesting that in some cases dimensional weighting can vary. Future work could examine whether amusics also rely less on pitch information when detecting musical structure. The boundaries of musical phrases, for example, are conveyed by pitch and durational changes similar to those found at the boundaries of linguistic phrases, including phrase-final lengthening and changes in pitch (Tierney, Russo, & Patel, 2011). Accordingly, typical listeners listen for both pitch and durational cues when judging the completeness of a musical phrase (Jusczyk & Krumhansl, 1993; Palmer & Krumhansl, 1987). Similarly, the location of musical beats can be conveyed by lengthening of the note on which the beat falls and/or a change in melodic contour (Ellis & Jones, 2009; Hannon et al., 2004; Prince, 2014). We predict that individual differences in dimensional salience (such as decreased pitch salience in amusia) extend across tasks and lead to measurable differences in perceptual strategies during beat and musical phrase perception.

Prior work has shown that relative weighting of pitch versus time cues can be modified by the presence of tonal and rhythmic structure (Prince & Rice, 2018; Prince, Schmuckler, & Thompson, 2009), suggesting that dimensional salience is not set in stone, but can be modified in response to cues to the relative usefulness of different sources of information. This suggests that dimensional weighting based on perceptual variance may not be limited to slow, long-term effects. Listeners may also dynamically alter their musical dimensional weighting in response to short-term changes in cue distribution, as has been shown for perception of phonetic features such as voice onset time (Holt & Lotto, 2006).

Our finding that amusics can minimize the impact of their perceptual impairment by focusing on preserved cues suggests that other populations may be able to take advantage of a similar strategy. Individuals with autism (O’Connor, 2012), ADHD (Riccio, Hynd, Cohen, Hall, & Molt, 1994), and beat deafness (Phillips-Silver et al., 2011), for example, have been reported to display impaired temporal but not pitch perception. Our model population was able to integrate pitch and duration together to perform the tasks, and this strategy may limit the impact of impaired auditory perception in some of these populations. Other groups, however, might find this strategy less successful; for instance, individuals with autism have difficulty integrating information across multiple senses (Marco, Hinkley, Hill, & Nagarajan, 2011).

In conclusion, our results showcase how widespread competency can hide individual differences in how individuals perceive the world. Perception can, on the surface, appear to be seamless and universal, with most people appearing to arrive at the same interpretations from the same information. This, however, can mask the true diversity of human experience.

## Supplementary Material

10.1037/xge0000688.supp
