# Supplementary material for: Tailored Perception: Individuals’ Speech and Music Perception Strategies Fit Their Perceptual Abilities
Source: J Exp Psychol Gen. 2019 Oct 7;149(5):914–34. doi: 10.1037/xge0000688 (PMC7133494; doi:10.1037/xge0000688)
Supplement: Supplementary file 1 [file xge0000688Supplement.docx]

**Supplementary Material**

**Section 1: Focus stimuli from Experiment 2**

| # | Start | Focused Word 1 | Focused Word 2 | Middle | Ending 1 | Ending 2 |
| --- | --- | --- | --- | --- | --- | --- |
| 1 | Mary likes to | read | books | but she doesn't like to | WRITE books | read MAGAZINES |
| 2 | Alice sometimes | pets | dogs | but she won't | WASH dogs | pet CATS |
| 3 | Bob has a | blue | shirt | but he doesn't have a | RED shirt | blue HAT |
| 4 | Claire has a | black | dress | but she doesn't have a | WHITE dress | black BAG |
| 5 | Dave likes to | study | music | but he doesn't like to | PLAY music | study HISTORY |
| 6 | Sally has a | Windows | computer | but she really wants | an APPLE computer | a Windows TABLET |
| 7 | George asked for a | white | Americano | but the barista gave him a | BLACK Americano | white filter COFFEE |
| 8 | Fiona was eating | strawberry | yoghurt | but she really wanted some | BLUEBERRY yoghurt | strawberry ICECREAM |
| 9 | Tom likes | barbecue | chicken | but not as much as | ROAST chicken | barebecue PORK |
| 10 | Sophie likes to | paint | landscapes | but she doesn’t like to | DRAW landscapes | paint PORTRAITS |
| 11 | John can't | run | a marathon | but he could | WALK a marathon | run a MILE |
| 12 | Matt is good at | flying | planes | but he isn't good at | LANDING planes | flying HELICOPTERS |
| 13 | Pippa found a | jam | jar | but she couldn't find a | JELLY jar | jam KNIFE |
| 14 | Sam has a | fish | knife | but he doesn't have a | BUTTER knife | fish FORK |
| 15 | Rachel likes | French | food | but she doesn't like | ITALIAN food | French WINE |
| 16 | The woman likes | white | pearls | but not | BLACK pearls | white DIAMONDS |
| 17 | Ken won't buy | Sainsbury's | pizza | but he will buy | TESCO'S pizza | Sainsbury's CHICKEN |
| 18 | Sarah has a | Barclay's | card | but she doesn't have a | LLOYDS card | Barclay's MORTGAGE |
| 19 | Neil won't support | Oxford's | fencing team | but he will support | CAMBRIDGE'S fencing team | Oxford's ROWING team |
| 20 | Carolyn likes | Scottish | pubs | but she doesn't like | ENGLISH pubs | Scottish RESTAURANTS |
| 21 | Micah has been to | Regent's | park | but he hasn't been to | HYDE Park | Regent's STREET |
| 22 | Rosalyn likes to | drink | beer | but she doesn't like to | BREW beer | drink LIQUOR |
| 23 | Veronica has visited | America | for holiday | but she hasn't visited | CANADA for holiday | America FOR WORK |
| 24 | Tim has an | electric | piano | but he really wants an | ACOUSTIC piano | electric GUITAR |
| 25 | Ben has ridden a | UK | train | but he has never ridden a | AMERICAN train | UK BUS |
| 26 | Nancy has a | small | flat | but she would really like a | LARGE flat | small HOUSE |
| 27 | Paul's house has a | brown | sofa | but it doesn't have a | BLACK sofa | brown CHAIR |
| 28 | Robert doesn't like | Dutch | cinema | but he does like | GERMAN cinema | Dutch THEATRE |
| 29 | Jenny doesn't have any | ginger | friends | but she does have several | BLONDE friends | ginger COLLEAGUES |
| 30 | You shouldn't open the | red | suitcase | but you can open the | GREEN suitcase | red CHEST |
| 31 | Emma doesn't | speak | well | but she does | DRESS well | speak OFTEN |
| 32 | Rose has visited | southern | Greece | but she has not visited | NORTHERN Greece | southern ITALY |
| 33 | Jane can speak | modern | Greek | but she can't speak | ANCIENT Greek | modern EGYPTIAN |
| 34 | Jim likes | Boots' | shampoo | but he doesn't like | SUPERDRUG shampoo | Boots' BODYWASH |
| 35 | Cameron will sometimes | watch | basketball | but he will never | PLAY basketball | watch CRICKET |
| 36 | Terry buys | sparkling | water | but not | STILL water | sparkling WINE |
| 37 | Richard said to buy | red | cups | but not | BLUE cups | red PLATES |
| 38 | Harriet can | speak | Mandarin | but she can't | READ Mandarin | speak CANTONESE |
| 39 | Olivia was looking for | wooden | boats | but she only found | PLASTIC boats | wooden PLANES |
| 40 | Michael likes to | plant | flowers | but he hates to | PICK flowers | plant POTATOES |
| 41 | Cathy likes to | observe | children | but she doesn't like to | TALK to children | observe ADULTS |
| 42 | Lily likes to | buy | stocks | but she doesn't like to | SELL stocks | buy BONDS |
| 43 | Alex likes to | collect | dolls | but he doesn't like to | PLAY with dolls | collect STAMPS |
| 44 | Frank has a | toy | dog | but he would really like a | REAL dog | toy BIRD |
| 45 | Nick likes to | sweep | kitchens | but he doesn't like to | MOP kitchens | sweep BATHROOMS |
| 46 | Bonnie has an | American | visa | but she really wants a | BRITISH visa | American PASSPORT |
| 47 | Patsy likes | Starbucks | coffee | but her friends like | COSTA coffee | Starbucks TEA |
| 48 | Timothy bought a | leather | jacket | because he couldn't find | a COTH jacket | leather SHOES |
| 49 | Carrie likes | Star Trek | films | but she can't stand | Star WARS films | Star TREK cartoons |
| 50 | Daniel enjoys | Chicago | pizza | but he doesn't care for | NEW YORK pizza | Chicago BEER |

**Section 2: Phrase Stimuli from Experiment 2**

| # | Start | Finish |
| --- | --- | --- |
| 1 | After Jane dusts, the dining table | is clean |
| 1 | After Jane dusts the dining table, | it's clean |
| 2 | After John runs, the race | is over |
| 2 | After John runs the race, | it's over |
| 5 | Because Mike phoned, his mother | was relieved |
| 5 | Because Mike phoned his mother, | she was relieved |
| 7 | Because Sarah answered, the teacher | was proud |
| 7 | Because Sarah answered the teacher, | she was proud |
| 8 | Because Tara cleaned, the house | was spotless |
| 8 | Because Tara cleaned the house, | it was spotless |
| 9 | Because George forgot, the party | had started |
| 9 | Because George forgot the party, | he was sad |
| 10 | Because Mike paid, the bill | was smaller |
| 10 | Because Mike paid the bill, | it was smaller |
| 13 | If Charles is baby-sitting, the children | are happy |
| 13 | If Charles is baby-sitting the children, | they're happy |
| 14 | If George is programming, the computer | is busy |
| 14 | If George is programming the computer, | it's busy |
| 15 | If Ian doesn't notice, Beth | is fine |
| 15 | If Ian doesn't notice Beth, | it's fine |
| 16 | If Joe starts, the meeting | will be long |
| 16 | If Joe starts the meeting, | it'll be long |
| 18 | If Laura is folding, the towels | will be neat |
| 18 | If Laura is folding the towels, | they'll be neat |
| 19 | When the baby finishes, the bottle | will be empty |
| 19 | When the baby finishes the bottle, | it'll be empty |
| 20 | If Barbara gives up, the ship | will be plundered |
| 20 | If Barbara gives up the ship, | it'll be plundered |
| 21 | If the Scissor Sisters open, the show | will be great |
| 21 | If the Scissor Sisters open the show, | it'll be great |
| 22 | If the maid packs, the suitcase | will be tidy |
| 22 | If the maid packs the suitcase, | it'll be tidy |
| 23 | If Tom wins, the contest | is over |
| 23 | If Tom wins the contest, | it's over |
| 24 | If the doctor calls, your sister | will answer |
| 24 | If the doctor calls your sister, | she'll answer |
| 25 | If Jack cleans, the kitchen | will be filthy |
| 25 | If Jack cleans the kitchen, | it'll be filthy |
| 26 | If dad digs, the hole | will be deep |
| 26 | If dad digs the hole, | it'll be deep |
| 27 | When a man cheats, his friends | get angry |
| 27 | When a man cheats his friends, | they're angry |
| 29 | When Gaga sings, the song | is a hit |
| 29 | When Gaga sings the song, | it's a hit |
| 30 | When Roger leaves, the house | is dark |
| 30 | When Roger leaves the house, | it's dark |
| 31 | When Suzie visits, her grandpa | is happy |
| 31 | When Suzie visits her grandpa, | he's happy |
| 32 | When the clock strikes, the hour | has started |
| 32 | When the clock strikes the hour, | it's started |
| 33 | When the guerrillas fight, the battle | has begun |
| 33 | When the guerrillas fight the battle, | it's begun |
| 34 | When the maid cleans, the rooms | are organized |
| 34 | When the maid cleans the rooms, | they're organized |
| 35 | When the original cast performs, the play | is fantastic |
| 35 | When the original cast performs the play, | it's fantastic |
| 36 | When Tim is presenting, the lectures | are interesting |
| 36 | When Tim is presenting the lectures, | they're interesting |
| 37 | When The Beatles play, the music | is noisy |
| 37 | When The Beatles play the music, | it's noisy |
| 38 | When Paul drinks, the rum | disappears |
| 38 | When Paul drinks the rum, | it disappears |
| 39 | When Mary helps, the homeless | are grateful |
| 39 | When Mary helps the homeless, | they're grateful |
| 40 | When the phone loads, the app | crashes |
| 40 | When the phone loads the app, | it crashes |
| 41 | When the shop closes, its doors | are locked |
| 41 | When the shop closes its doors, | they're locked |
| 42 | When a train passes, the station | shakes |
| 42 | When a train passes the station, | it shakes |
| 43 | When the actor practices, the monologue | is excellent |
| 43 | When the actor practices the monologue, | it's excellent |
| 44 | When the cowboy rides, the horse | is tired |
| 44 | When the cowboy rides the horse, | it's tired |
| 46 | Whenever the guard checks, the door | is locked |
| 46 | Whenever the guard checks the door, | it's locked |
| 47 | Whenever Bill teaches, the course | is boring |
| 47 | Whenever Bill teaches the course, | it's boring |
| 48 | Whenever a customer tips, the waiter | is pleased |
| 48 | Whenever a customer tips the waiter, | he's pleased |
| 49 | Whenever Rachel leads, the discussion | is exciting |
| 49 | Whenever Rachel leads the discussion, | it's exciting |
| 50 | Whenever Mary writes, the paper | is excellent |
| 50 | Whenever Mary writes the paper, | it's excllent |

**
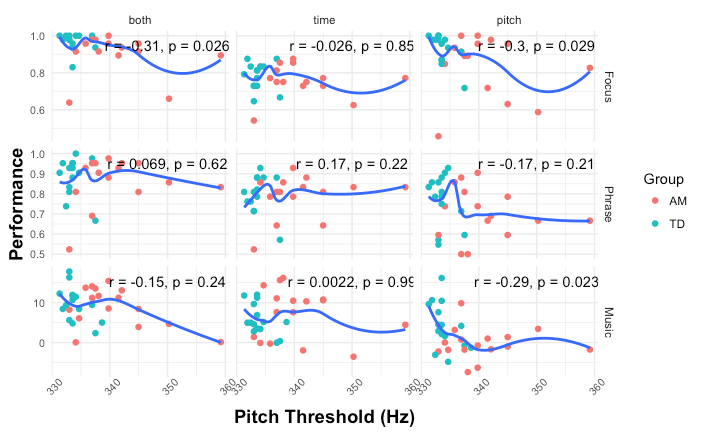
**

**Figure S1 Performance on the Focus, Linguistic Phrase and Musical Phrase tests predicted by pitch thresholds.** Because non-parametric statistics were used in the correlations, LOESS was used to fit lines showing direction of trends.

**
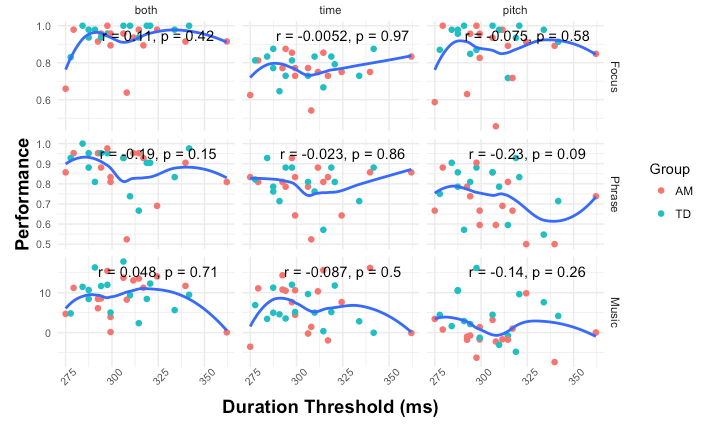
**

**Figure S2. Performance on the Focus, Linguistic Phrase and Musical Phrase tests predicted by duration thresholds.**

**
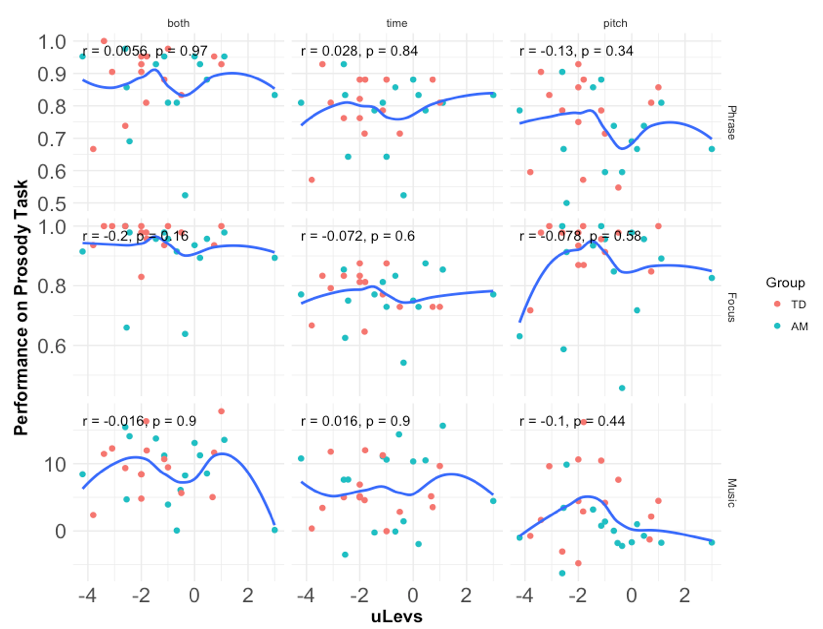
**

**Figure S3. Performance on the Focus, Linguistic Phrase and Musical Phrase tests predicted by Speech in Noise thresholds.**

**Table S1: Musical Phrase Test, all pairwise contrasts (p-values FDR-adjusted). Estimate = difference in rating. SE = standard error. df = degrees of freedom.**

| **Condition** | **Group** | **Contrast** | **Estimate** | **SE** | **df** | **T** | **p** |
| --- | --- | --- | --- | --- | --- | --- | --- |
| Combined | ~ | CONT vs AMUS | 0.018 | 0.035 | 119.93 | 0.52 | 0.611 |
| Duration | ~ | CONT vs AMUS | -0.025 | 0.035 | 119.93 | -0.71 | 0.611 |
| Pitch | ~ | CONT vs AMUS | 0.099 | 0.035 | 119.93 | **2.87** | **0.022** |
| ~ | CONT | Combined vs Duration | 0.079 | 0.062 | 192.94 | 1.27 | 0.370 |
| ~ | CONT | Combined vs Pitch | 0.110 | 0.062 | 192.94 | 1.78 | 0.173 |
| ~ | CONT | Duration vs Pitch | 0.032 | 0.062 | 192.94 | 0.509 | 0.661 |
| ~ | AMUS | Combined vs Duration | 0.036 | 0.062 | 187.06 | 0.588 | 0.611 |
| ~ | AMUS | Combined vs Pitch | 0.192 | 0.062 | 187.06 | **3.115** | **0.019** |
| ~ | AMUS | Duration vs Pitch | 0.156 | 0.062 | 187.06 | **2.53** | **0.037** |
| ~ | ALL | Combined vs Duration | 0.058 | 0.058 | 147.04 | 0.993 | 0.323 |
| ~ | ALL | Combined vs Pitch | 0.151 | 0.058 | 147.04 | **2.607** | **0.030** |
| ~ | ALL | Duration vs Pitch | 0.094 | 0.058 | 147.04 | 1.614 | 0.163 |

**Table S2: Linguistic Focus test: pairwise comparisons of marginal means (p-values FDR adjusted). OR = odds ratio. SE = standard error.**

| **Condition** | **Group** | **Contrast** | **OR** | **SE** | | **Z** | | **p** |
| --- | --- | --- | --- | --- | --- | --- | --- | --- |
| Combined | ~ | CONT vs AMUS | 2.44 | 0.13 | **2.71** | | **0.009** |  |
| Duration | ~ | CONT vs AMUS | 1.11 | 0.24 | 0.39 | | 0.697 |  |
| F0 | ~ | CONT vs AMUS | 2.00 | 0.14 | **2.39** | | **0.019** |  |
| ~ | AMUS | Combined vs F0 | 2.06 | 0.37 | **4.01** | | **<.001** |  |
| ~ | AMUS | Combined vs Duration | 3.71 | 0.64 | **7.56** | | **<.001** |  |
| ~ | AMUS | F0 vs Duration | 1.80 | 0.28 | **3.83** | | **<.001** |  |
| ~ | CONT | Combined vs F0 | 2.52 | 0.62 | **3.77** | | **<.001** |  |
| ~ | CONT | Combined vs Duration | 8.15 | 1.84 | **9.31** | | **<.001** |  |
| ~ | CONT | F0 vs Duration | 3.23 | 0.57 | **6.65** | | **<.001** |  |
| ~ | ALL | Combined vs F0 | 2.28 | 0.36 | **5.22** | | **<.001** |  |
| ~ | ALL | Combined vs Duration | 5.50 | 0.82 | **11.44** | | **<.001** |  |
| ~ | ALL | F0 vs Duration | 2.41 | 0.30 | **7.10** | | **<.001** |  |

**Table S3: Post hoc contrasts, Linguistic Phrase Test**

| **Condition** | **Group** | **Contrast** | **OR** | **SE** | **Z** | **P** |
| --- | --- | --- | --- | --- | --- | --- |
| Combined | ~ | CONT vs AMUS | 1.10 | 0.26 | 0.32 | 0.841 |
| Duration | ~ | CONT vs AMUS | 1.01 | 0.27 | 0.02 | 0.985 |
| F0 | ~ | CONT vs AMUS | 1.35 | 0.20 | 1.12 | 0.338 |
| ~ | AMUS | Combined vs F) | 2.88 | 0.44 | **7.00** | **<0.001** |
| ~ | AMUS | Combined vs Duration | 1.77 | 0.28 | **3.64** | **0.001** |
| ~ | AMUS | Duration vs Pitch | 1.63 | 0.08 | **3.56** | **0.001** |
| ~ | CONT | Combined vs F0 | 2.34 | 0.37 | **5.37** | **<0.001** |
| ~ | CONT | Combined vs Duration | 1.93 | 0.31 | **4.10** | **<0.001** |
| ~ | CONT | Duration vs F0 | 1.21 | 0.12 | 1.34 | 0.268 |
| ~ | ALL | Combined vs F0 | 2.60 | 0.28 | **8.71** | **<.001** |
| ~ | ALL | Combined vs Duration | 1.85 | 0.21 | **5.48** | **<.001** |
| ~ | ALL | Duration vs F0 | 1.41 | 0.07 | **3.43** | **<.001** |

**Table S4: T-statistics for results of Experiment 1 – Phonetic Task**

|  | **VOT-1** | **VOT-2** | **VOT-3** | **VOT-4** | **VOT-5** |
| --- | --- | --- | --- | --- | --- |
| **F0-1** | 0.26017 | 0.59554 | 1.4955 | 0.74754 | 1 |
| **F0-2** | 0.60937 | 0.43728 | 0.56653 | 0.57394 | 0.76632 |
| **F0-3** | 0.61276 | 0.15215 | -0.64343 | -1.0496 | 0.51441 |
| **F0-4** | -0.3322 | -0.64718 | -0.394 | 0.24218 | 0.36585 |
| **F0-5** | -0.98711 | 1.7889 | -1.6134 | -1.6197 | -1.6641 |

**Table S5: T-statistics for results of Experiment 1 – Prosodic Task**

|  | **Dur-1** | **Dur-2** | **Dur-3** | **Dur-4** | **Dur-5** | **Dur-6** | **Dur-7** |
| --- | --- | --- | --- | --- | --- | --- | --- |
| **F0-1** | 4.222 | 3.608 | 2.9405 | 3.6179 | 2.1974 | 2.8942 | 1.745 |
| **F0-2** | 4.7326 | 4.3493 | 3.5842 | 3.7018 | 3.0025 | 2.4465 | 2.7616 |
| **F0-3** | 3.6108 | 3.8616 | 5.1845 | 2.6749 | 3.3535 | 1.8273 | 1.8869 |
| **F0-4** | 1.5283 | 4.0606 | 2.3548 | 2.2862 | 0.92245 | 1.7273 | 0.71722 |
| **F0-5** | 0.63427 | 0.94281 | 0.41478 | 0.18732 | 0.22835 | -1.3163 | -0.94812 |
| **F0-6** | -1.539 | -0.59761 | -0.21719 | -1.4914 | -1.4841 | -1.8605 | -2.0126 |
| **F0-7** | -0.53074 | 0.76696 | -1.8752 | -2.3814 | -1.822 | -2.2963 | -1.9654 |
